# Dynamics of Temporal Integration in the Lateral Geniculate Nucleus

**DOI:** 10.1523/ENEURO.0088-22.2022

**Published:** 2022-08-23

**Authors:** Prescott C. Alexander, Henry J. Alitto, Tucker G. Fisher, Daniel L. Rathbun, Theodore G. Weyand, W. Martin Usrey

**Affiliations:** 1 Center for Neuroscience, University of California, Davis, Davis, CA 95616; 2 Center for Vision Science, University of California, Davis, Davis, CA; 3 Department of Neurobiology, Physiology, and Behavior, University of California, Davis, Davis, CA 95616; 4 Department of Neurobiology, Stanford University School of Medicine, Stanford, CA 94305; 5 Department of Ophthalmology, Henry Ford Health System, Detroit, MI 48202; 6 Department of Cell Biology and Anatomy, Louisiana State University Health Sciences Center, New Orleans, LA 70112

**Keywords:** coding, generalized linear models, LGN, retina, synapse, vision

## Abstract

Before visual information from the retina reaches primary visual cortex (V1), it is dynamically filtered by the lateral geniculate nucleus (LGN) of the thalamus, the first location within the visual hierarchy at which nonretinal structures can significantly influence visual processing. To explore the form and dynamics of geniculate filtering we used data from monosynpatically connected pairs of retinal ganglion cells (RGCs) and LGN relay cells in the cat that, under anesthetized conditions, were stimulated with binary white noise and/or drifting sine-wave gratings to train models of increasing complexity to predict which RGC spikes were relayed to cortex, what we call “relay status.” In addition, we analyze and compare a smaller dataset recorded in the awake state to assess how anesthesia might influence our results. Consistent with previous work, we find that the preceding retinal interspike interval (ISI) is the primary determinate of relay status with only modest contributions from longer patterns of retinal spikes. Including the prior activity of the LGN cell further improved model predictions, primarily by indicating epochs of geniculate burst activity in recordings made under anesthesia, and by allowing the model to capture gain control-like behavior within the awake LGN. Using the same modeling framework, we further demonstrate that the form of geniculate filtering changes according to the level of activity within the early visual circuit under certain stimulus conditions. This finding suggests a candidate mechanism by which a stimulus specific form of gain control may operate within the LGN.

## Significance Statement

The lateral geniculate nucleus (LGN) is a dynamic, tunable filter, transforming information as it flows from the retina to primary visual cortex (V1). In this work we use a large dataset of monosynaptically connected retinal ganglion cell (RGC) and LGN cell pairs to model the filtering function performed by individual LGN neurons in the anesthetized or awake state. We demonstrate that, while much of the filtering that the LGN performs can be accounted for by temporal summation, other factors, such as the bursting activity of relay cells, also play a role. Additionally, we show that the time scale of summation is dynamic under certain stimulus and network conditions and that the integration dynamics are largely similar between the anesthetized and awake states.

## Introduction

There are two primary dimensions along which relay cells of the lateral geniculate nucleus (LGN) might transform the visual information that they receive from the retina, namely, space and time. In the spatial dimensions, a substantial body of evidence suggests a limited transformation, most notably an increase in the strength of the antagonistic surround of the center/surround receptive field (RF; Usrey et al., 1998, 1999; Wang et al., 2010). On the other hand, data demonstrating substantial temporal transformations by LGN relay cells of their direct retinal inputs abound (Usrey et al., 1998; Carandini et al., 2007; Sincich et al., 2007, 2009; Babadi et al., 2010; Wang et al., 2010; Rathbun et al., 2016). Prior work has demonstrated that the temporal transformation performed by the LGN results in an increased encoding efficiency in the signals sent by the LGN to primary visual cortex (V1) compared with the signals received from the retina (Sincich et al., 2009; Uglesich et al., 2009; Wang et al., 2010) and that this increased efficiency can be explained by temporal summation within relay cells (Carandini et al., 2007; Sincich et al., 2007; Casti et al., 2008) and a selective filtering out of less informative retinal spikes (Rathbun et al., 2010). Furthermore, it has recently been shown that temporal summation within the LGN changes with stimulus contrast (Alitto et al., 2019a), suggesting that geniculate filtering is dynamic and can adapt to the statistics of the visual environment. The aim of this work is to investigate this filtering process by modeling the input-output relation of LGN cells using generalized linear models (GLMs), and to further examine whether the input-output relation changes under different stimulus or network conditions.

In order to investigate the input-output relation of LGN relay cells, we first assembled a large database of simultaneous, extracellular recordings of monosynaptically connected retinal ganglion cell (RGC)-LGN cell pairs from previously published work in anesthetized cats (Usrey et al., 1998; Rathbun et al., 2010; Fisher et al., 2017). Although these data offer a near optimal level of spatial and temporal resolution with which to examine input-output relations in single neurons, they only capture a single RGC input to each relay cell, which are thought to receive input from between two and five RGCs in the cat (Cleland et al., 1971; Usrey et al., 1999). Thus, instead of focusing our analyses on the full spike train produced by relay cells, which contains contributions from all RGC inputs, we instead focus specifically on trying to model the process that determines which spikes from the recorded RGC are relayed, that is elicit a spike in their geniculate partner, and which are not. We begin by considering the simplest model of temporal summation, the often used interspike interval (ISI) model (Usrey et al., 1998; Sincich et al., 2007; Weyand, 2007; Wang et al., 2010; Rathbun et al., 2016; Alitto et al., 2019a) whereby the relay probability of each retinal spike is predicted based on the elapsed time since the last retinal spike. We then show how the ISI model can be conceptually extended using GLMs, allowing the full pattern of retinal spikes, within a given window of time, to be used in the predictions. We then introduce a two component GLM that includes the pattern of LGN spikes preceding each retinal spike to investigate whether the LGN spike train contains additional information about the relay probability of future retinal spikes. Finally, we explore whether high levels of activity within the retino-thalamo-cortical circuit influences how LGN relay cells integrate their retinal inputs, and whether this change might explain the dynamic temporal filtering within relay cells that has been previously reported (Rathbun et al., 2016; Alitto et al., 2019a).

While this approach allows the computations performed by individual LGN relay cells to be examined with a level of detail unmatched by any other existing method, it does require anesthesia to record the spiking activity of individual RGCs within the eye. In order to complement this approach, and to offer more general findings, we additionally analyze a smaller dataset recorded from awake cats in which S-potentials, the extracellular record of excitatory postsynaptic potentials driven by the dominant retinal input (Kaplan and Shapley, 1984), were recorded simultaneously with the LGN spikes that they frequently elicit (Weyand, 2007). Given the small size of the awake dataset, we cannot make quantitative comparisons between the anesthetized and awake state. However, we can use the awake dataset to qualitatively confirm, or refute, whether our findings from the anesthetized state are generally applicable.

## Materials and Methods

### Data sources

The data analyzed in this study contributed to previous reports on the retinogeniculate pathway in both anesthetized (Usrey et al., 1998, 1999; Rathbun et al., 2010, 2016; Fisher et al., 2017; Alitto et al., 2019b) and awake (Weyand, 2007) cats. All experimental procedures conformed to National Institutes of Health and United States Department of Agriculture guidelines and were approved by the Institutional Animal Care and Use Committee at the University of California, Davis or Louisiana State University Health Sciences Center.

### Code accessibility

All data and code used in this study are available at https://github.com/scottiealexander/relayglm_paper. The code is also available as Extended Data.

#### Computing and software resources

All analyses were performed on a Dell Precision T3610 desktop with an Intel Xenon processor (E5-1620) running the Lubuntu 18.04.6 operating system.

All analyses were performed using custom written code in the Julia programming language version 1.6.1 (Bezanson et al., 2017). Visualizations were created using the Julia interface to the Matplotlib graphics package (Hunter, 2007).

### Anesthetized recordings

#### Surgery and preparation

Twenty-three adult cats of either sex contributed to this dataset. As previously described, anesthesia was initiated with ketamine (10 mg/kg, i.m.) or ketamine and thiopental sodium (20 mg/kg, i.v.) and maintained with either sodium pentothal (2–3 mg/kg/h, i.v.), or isoflurane (0.7–2%). Administration rate of the anesthetic agent was increased when physiological monitoring indicated low levels of anesthesia. A tracheotomy was performed and animals were placed in a stereotaxic apparatus and mechanically respired. Body temperature, ECG, EEG, and expired CO_2_ were monitored for the duration of the experiment. All wound margins were infused with lidocaine. The cortical surface overlying the LGN was exposed by a craniotomy and durotomy and then protected with a layer of agarose. To minimize eye movements and facilitate retinal recordings, the sclera beneath the lateral margin of each eye was glued to a rigid ring that was mounted to the stereotaxic frame. The posterior chamber of each eye was accessed via a trans-scleral guide tube inserted through the ring. Upon completion of surgical procedures, animals were paralyzed with either vecuronium bromide (0.2 mg/kg/h, i.v.) or gallium triethiodide (6–8 mg/kg/h). The nictitating membranes of the eye were retracted with 10% phenylephrine and pupils were maintained in a dilated state with 1% atropine sulfate and flurbiprofen sodium (1.5 mg/h). The eyes were then refracted, fitted with contact lenses, and focused on a tangent screen in front of the animal.

#### Electrophysiological recording and visual stimuli

Extracellular recordings of RGCs were made using single, parylene-coated microelectrodes (AM Systems) inserted through the trans-scleral guide tube into the posterior chamber of the eye via a custom-made manipulator. Extracellular recordings of LGN cells in the A laminae were made using a seven-channel multielectrode array (Thomas Recording). Neural signals were amplified, filtered (AM Systems, Thomas Recording) and recorded by either a computer running Brainwave software (Datawave Systems) or a 1401 data acquisition system connected to a computer running the Spike2 software package (Cambridge Electronic Design). Single-neuron isolation was based on waveform analysis and the presence of a refractory period in the auto-correlogram.

Visual stimuli were generated by either a Pepper Graphics System video card (Number Nine Computer Corporation) and presented on a CRT monitor at 80 or 100 Hz (NEC Multisync), or a VSG 2/5 visual stimulus generator (Cambridge Research Systems) and presented on a γ-calibrated CRT monitor at 140 Hz (Sony). Drifting sinewave gratings that varied in either contrast or diameter were presented at a temporal frequency of 4 Hz and at the optimal spatial frequency for the RGC-LGN pair under study. Binary white-noise stimuli were comprised of a 16 × 16 grid of squares where the brightness of each square (black or white) on each stimulus frame was governed by a 2^15^–1 frame long pseudorandom sequence (the “m-sequence”; Sutter, 1987; Reid et al., 1997). The stimulus frame was updated either on every or every other monitor frame (7- to 25-ms stimulus frame duration).

### Awake recordings

Four adult cats of either sex contributed to this dataset. Details of surgical procedures, training, and recording have been described previously (Weyand and Gafka, 1998; Weyand, 2007). In brief, animals underwent an initial implant surgery to allow for head-fixed training and eye tracking, followed by a training period in which animals learned to maintain fixation to within 1.5° of a small spot (0.2°) for 1–3 s to receive a food reward. Following the training period animals underwent a second surgery in which a canula was introduced into the brain (∼6 mm deep) through a small craniotomy and fixed in place allowing a microelectrode to access the LGN for awake, extracellular, recordings (the orientation of the canula could be adjusted; for details, see Weyand, 2007). Signals from microelectrodes (1–1.5 MΩ at 1 kHz) were amplified (100–1000×), filtered (0.001–10 kHz), and digitized at 22.5 kHz by a modified VCR (A. R. Vetter) and transferred to a computer for storage using hardware and software from National Instruments. S-potentials and action potentials were identified and sorted offline using Mini-Analysis (for details, see Weyand, 2007). As S-potentials are thought to be the extracellular record of excitatory postsynaptic potentials driven by the dominant retinal input (Cleland et al., 1971; Kaplan and Shapley, 1984; Weyand, 2007), the delay between a successful S-potential (reflecting a relayed RGC spike) and the triggered LGN spike is substantially shorter than the analogous delay between an RGC spike recorded within the eye and the LGN spike that it triggers. Thus, for the analyses presented in this paper the timing of the S-potentials for a given pair were shifted “backwards” in time relative to the paired LGN spikes by 2.4 ms which ensured that the median delay between S-potentials and triggered LGN spikes (which was 0.4 ms before shifting) matched the median delay observed between RGC and triggered LGN spikes in the anesthetized dataset (2.8 ms). This shift helps to minimize any contribution from the different recording approaches to any differences in timing that may be observed between the awake and anesthetized datasets and allows S-potentials to be identified as relayed or not using the same criteria as those used for RGC spikes recorded within the eye (see below, Identification of monosynaptically connected pairs and relayed RGC spikes). For simplicity, throughout this paper we refer to both RGC spikes recorded within the eye as well as time-shifted S-potentials as “RGC spikes.”

Given the difficulty of recording S-potentials in an awake animal, the duration over which individual pairs could be recorded was often quite limited and thus most of the pairs analyzed in this study (seven of eight) were not presented with a controlled stimulus but were instead stimulated by whatever features of the well-lit room fell within their RF (for details, see Weyand, 2007). The one exception, pair 200001250, was stimulated with a sinewave grating (see Extended Data Fig. 7-2).

### Data analysis

#### Identification of monosynaptically connected pairs and relayed RGC spikes

Simultaneously recorded RGC and LGN cells that showed a prominent, short-latency peak in their spike time cross-correlograms were considered to be monosynaptically connected pairs (Mastronarde, 1987; Usrey et al., 1999; Rathbun et al., 2010; Fisher et al., 2017). All S-potential-LGN pairs from Weyand (2007) met this criterion by definition. Cross-correlograms, LGN spiking relative to each RGC spike, were constructed for all pairs (from both the anesthetized and awake datasets) using 0.1-ms bins. Peaks were considered prominent if at least one bin exceeded a threshold of μ_baseline_ + 3 σ_baseline_, where μ_baseline_ is the mean of the baseline period spanning 30–50 ms on either side of the peak bin, and σ_baseline_ is the standard deviation (SD) of the baseline period. All bins adjacent to the peak bin that also exceeded the threshold were considered part of the peak. Peaks were considered short latency if they occurred within 2–6 ms of t= 0, the time of each retinal spike. All retinal spikes that were followed by a LGN spike that fell within the peak bins of cross-correlograms were considered “relayed,” all other retinal spikes were considered “nonrelayed.” Retinal efficacy (or simply efficacy) is the number of relayed spikes divided by the total number of retinal spikes. Likewise, all LGN spikes that were preceded by a retinal spike within the monosynaptic window (defined as above) were considered “triggered.” Retinal contribution (or simply contribution) is the number of triggered LGN spikes divided by the total number of LGN spikes.

#### Modeling framework

All models discussed in the paper generally take the form:

(1) λ=σ(f(t|θ)),

where t are retinal spike times, θ are the model parameters, and λ are the predicted relay probabilities in (0, 1). For the ISI model, f is a nonlinear map between the interval t_i_ − t_i−1_ and a conditional intensity. For GLMs, f is a linear function of t represented as a binary vector over an n millisecond interval before each t_i_. For two component GLMs, f also takes as input the LGN spike times t_LGN_, f ( t; t_LGN_| θ). For all models, σ is the logistic function:

(2) $$\sigma (x)=\frac{1}{1+\exp(-x)},$$

which maps a conditional intensity to a relay probability.

#### Assessing model performance

All models were assessed in a train-on-90%, test-on-10% 10-fold cross-validation procedure. In each fold, 90% of the data were used to fit the model and the remaining 10% was used only to assess model performance. This procedure was performed ten times such that all data appear in the test set exactly once. Data partitioning across folds was performed such that all test sets contained approximately the same number of relayed spikes. This balancing helped reduce the variability in mean efficacy across folds for a given pair, which serves to stabilize the performance metric that we used (see below) especially for pairs with relatively low mean efficacy. Model performance is the mean performance across folds.

As all models presented in this paper produce a relay probability (0,1) for each retinal spike, we use the cross-validated single-event Bernoulli information ( I_Bernoulli_) to assess model performance. I_Bernoulli_ is the Bernoulli analog of the cross-validated single-spike information used for Poisson GLMs (Williamson et al., 2015) and can be calculated from the Bernoulli log-likelihood function L (Truccolo et al., 2005; Williamson et al., 2015):

(3) L(λ;y(t))=∑y(t)log(λ) + (1−y(t))log(1−λ),

where λ are the predicted relay probabilities (as above), and y(t) indicates whether each retinal spike was relayed as {0,1}, what we call “relay status.” Using L we can calculate $I_{\text{Bernoulli}}$:

(4) $$I_{\text{Bernoulli}}=\frac{1}{n_{\text{test}}\log(2)}\left(L\left(\lambda_{\text{train}};y_{\text{test}}\right)-L\left(y_{\text{test}}\right)\right),$$

where λ_train_ = λ( t_test_| θ_train_) are the predicted relay probabilities for test-set retinal spikes ( t_test_) given the parameters ( θ_train_) learned from the training-set. y_test_ = y( t_test_) is the observed relay status for t_test_, and n_test_ = length( t_test_) is the number of retinal spikes in t_test_. L( y_test_) represents the log-likelihood of a homogeneous Bernoulli model where the mean efficacy of the test-set is predicted for every spike:

(5) $$L(y_{\text{test}})=r_{\text{test}}\;\text{log}({\bar{\lambda }}_{\text{test}})+(n_{\text{test}}-r_{\text{test}})\;\text{log}\;(\text{1}-{\bar{\lambda }}_{\text{test}}),$$

where r_test_ = ∑ y_test_ and $\bar{\lambda }$_test_ is the mean efficacy across t_test_, i.e., $\frac{r_{\text{test}}}{n_{\text{test}}}$).

In this construction, I_Bernoulli_ has units of bits/spike and for well fit models will take on values between ∼0 (no better than a homogeneous model) and 1 (perfect performance). In practice, poorly fit models can result in negative I_Bernoulli_ because of separate training and testing datasets (i.e., cross-validation). Conceptually, I_Bernoulli_ quantifies how informative model predictions are about the relay status of the test-set relative to a homogeneous model with the same mean efficacy as the test-set. While somewhat elaborate compared with metrics like accuracy, for a binary process like relay status it is important to take into account the fact that as $\bar{\lambda }$_test_ approaches 0 or 1, correctly predicting the outcome becomes trivial. Quantifying model performance relative to a homogeneous model ensures that as $\bar{\lambda }$_test_ approaches 0 or 1, I_Bernoulli_ approaches 0 for a model with perfect predictions (and values<0 for lesser performing models). While this behavior is necessary to accurately quantify model performance on this kind of classification task (i.e., where the number of relayed and nonrelayed spike cannot be matched), it entails that the maximum achievable I_Bernoulli_ depends in part on the mean efficacy of the RGC-LGN pair being modeled. For example, for a pair with an efficacy of 0.05 the maximum I_Bernoulli_ for a perfect performing model is 0.286 bits/spike.

#### ISI models

Usrey et al. (1998) described the effect of retinal ISI on efficacy, using the term “paired spike enhancement.” They observed that retinal spikes following short ISIs have a higher efficacy than those following long ISIs. Following (Wang et al., 2010), we recast that observation as a simple model for predicting which retinal spikes were relayed based on the elapsed time since the last retinal spike. This model was constructed by creating a histogram of the ISIs preceding all relayed retinal spikes and dividing the count in each bin by the total number retinal ISIs that fell within that bin. We used a bin width of 1 ms and the resulting histograms were smoothed with a unit-area Gaussian (the SD of which was chosen separately for each pair; see below, Hyperparameter optimization) to produce a function relating ISI to efficacy (ISI-efficacy function) which we denote as P ( t| t_ISI_) where t is the time of a retinal spike and t_ISI_ = t_i_ − t_i−1_ is the ISI preceding t for ISIs up to a maximum ( ISI_MAX_) that was chosen separately for each pair (see below, Hyperparameter optimization). For any retinal spikes with ISIs greater than ISI_MAX_, the model predicted the average efficacy across all ISIs in the corresponding dataset. For example, if the ISI of a retinal spike within a given test-set is greater than ISI_MAX_, the model would predict the mean efficacy of that test-set (i.e., $\frac{r_{\text{test}}}{n_{\text{test}}}$).

After building P ( t| t_ISI_) (abbreviated as P below for clarity) for a given pair, the fitting algorithm then found a linear transform f(P)=βP + α such that the Bernoulli log-likelihood of the resulting predictions

(6) $$\lambda_{\text{ISI}}=\sigma (f(P))$$

was maximized. This allows a shifting and rescaling of the predictions such that the mean of λ_ISI_ matches the mean efficacy of the data being used for model fitting. Omitting this step would penalize the ISI model quite significantly in the calculation of I_Bernoulli_ because λ_ISI_ may be incorrectly scaled relative to the mean efficacy (because of the ISI cutoff) and thus the likelihood of the homogeneous model, L( λ) above, would be expected to be large relative to L( λ_ISI_), yielding potentially negative values for I_Bernoulli_ that would incorrectly indicate poor performance.

As with all models discussed herein, for quantifying performance all parameters and hyperparameters were determined from an independent subset of the data from that used to assess performance (see above, Assessing model performance).

#### GLMs

##### General

In order to generalize the ISI based model to consider all activity within a period of time preceding each retinal spike, we used a GLM framework (Truccolo et al., 2005; Paninski et al., 2007; Pillow et al., 2008; Babadi et al., 2010). GLMs are a generalization of ordinary linear regression in which the to-be-predicted, or “response,” variable need not be normally distributed, and the predictor variables and response variable need not be linearly related (Nelder and Wedderburn, 1972). Similarly, GLMs can be thought of as a particular class of linear-nonlinear (LN) cascade models in which the nonlinearity, or activation function, is fixed and invertible (Chichilnisky, 2001; Paninski, 2004). GLMs generally take the form:

(7) y=g(Xθ),

where y is the response variable, X is a matrix of predictors, θ is a vector of model parameters, and g is the activation function (formally, the inverse link function). Given an assumed or known error distribution of y and an appropriate choice of g, the parameters θ can be efficiently fit by maximum likelihood methods (Paninski, 2004; Babadi et al., 2010).

In the present context, the response ( y) that we are trying to predict is the (binary) relay status of each retinal spike. Thus, a natural choice for the error distribution of y is the Bernoulli distribution, and a natural choice of activation function is the logistic function (i.e., logistic regression). The Bernoulli-Logistic GLM is given by:

(8) y=λ(t|θ)=σ(Xθ),

where t are the retinal spike times, and the predictor matrix X is derived from the retinal spike times alone [retinal history (RH) model] or using both the retinal and LGN spikes times [combined history (CH) model]. The relay status, y, of a set of retinal spikes, t, is then modeled as:

(9) y(t)∼Bernoulli(λ(t|θ)).

The parameter vector θ that minimized the negative log-likelihood (i.e., − L) for each model instance was found using Newton’s method (Nocedal and Wright, 2006) as implemented in (Mogensen and Riseth, 2018).

##### RH models

Within the GLM framework used here, X is an m by n + 1 matrix where m is the number of retinal spikes being used to fit the model (typically 90% of the retinal spikes recorded under a given stimulus condition, see above, Assessing model performance) and n is the number of temporal components. The additional column is the additive offset or “y-intercept” term. In the “RH only” version (RH) of the model, whose predictor matrix, sans-offset, we will refer to as X_R_, the “temporal components” are simply n 1-ms time bins representing the retinal spike train, as a binary vector, during the n milliseconds preceding each retinal spike. In this form, summing over the m rows of X_R_ would yield the autocorrelogram of the retinal spike train over an n millisecond window. The hyperparameter n was optimized separately for each pair (see below, Hyperparameter optimization). The n parameters corresponding to the n time bins of a fitted model can be thought of as a linear kernel or filter that reflects the extent to which retinal spikes occurring at a given time before the “target spike” influence the likelihood that the target spike will be relayed.

Given previous work suggesting that LGN temporal filters are likely to be smooth functions in this context (Usrey et al., 1998; Rathbun et al., 2010), and to help prevent overfitting, we introduce a smoothing prior on θ (excluding the y-intercept term), yielding a maximum a posteriori (MAP) estimator for θ:

(10) $$L_{\text{MAP}}\left(\theta \right)=L\left(\theta \right)-\eta \sum {\left(\theta_{\text{i}}-\theta_{\text{i}-\text{1}}\right)}^{\text{2}},$$

where the prior weighting term η is optimized separately for each pair (see below, Hyperparameter optimization).

##### CH models

The CH model extends the RH model by introducing another set of predictors derived from the activity of the LGN cell. In the RH model, the LGN cell only contributes by classifying each retinal spike as relayed or nonrelayed, whereas in the CH model the recent activity of the LGN cell can also contribute to the relay prediction (via spikes not “caused” by the recorded RGC). For the CH model, the predictor matrix X_C_ can be thought of as the column-wise concatenation of X_R_ with an analogous binary matrix, X_L_, of size m by p where each row of length p is the LGN cell’s binary spike train (1-ms bin size) during the p milliseconds preceding each retinal spike. Thus, summing over the rows of X_L_ would yield the cross-correlogram of the LGN activity relative to the RGC spike times for negative time lags. Importantly, the time window in which the LGN cell could respond to a given RGC spike was not included; the model could only consider events preceding a retinal spike in predicting whether or not it was relayed.

To help prevent overfitting we introduce a Gaussian prior on the coefficients of the CH model ( θ_C_) to penalize large coefficient values (i.e., ridge regression), yielding a MAP estimator for θ_C_:

(11) $$L_{\text{MAP}}(\theta_{\text{C}})=L(\theta_{\text{C}})-\eta \sum \theta_{\text{C}}{}^{\text{2}}$$

where, as above, η is optimized separately for each pair (see below, Hyperparameter optimization).

As the CH model is an extension of the RH model, the time window spanned by X_R_ was fixed, for each pair, at the value derived from RH model fitting (see above, RH models). The time window spanned by X_L_ was optimized for each pair in an analogous manner (see below, Hyperparameter optimization).

In order to help mitigate the cost of increasing the number of free parameters (which would otherwise increase quite dramatically), for CH models X_R_ and X_L_ were represented in a basis of raised-cosine functions following common practice (Pillow et al., 2005, 2008; Ghanbari et al., 2017):

(12) $$b_{k}\left(t\right)=\frac{\cos\left(q_{k}\left(t\right)\right)+1}{2}$$

(13) $$q_{k}\left(t\right)=(\log\left(t+\Psi \right)-\log\left(\phi_{k}+\Psi \right))\frac{\pi }{2\gamma },$$

such that $q_{\text{k}}\left(t\right)\in [-\pi ,\pi ]$, where $\phi_{\text{k}}$ is the center of the “raised bump” of the k-th basis vector, Ψ is a constant hyperparameter (see below, Hyperparameter optimization) that controls the linearity of the spacing between bumps, and γ is a scaling factor that controls the width of the bumps such that they tile the time axis (i.e., γ is a function of the number of basis vectors and the duration they need to cover). This representation greatly reduces the number of parameters while still allowing good temporal resolution around the time of the retinal spike (by setting Ψ closer to ∼1) at the cost of forcing the kernels to be smooth. However, this smoothness assumption is well supported by Usrey et al. (1998) and Rathbun et al. (2010) and loosely resembles, in its effects, the smoothing prior used in fitting RH models.

In practice X_C_ is a m by n_R_ + n_L_ + 1 matrix where n_R_ and n_L_ are the number of basis vectors used to represent X_R_ and X_L_, respectively. Here, n_R_ was set to 16 and n_L_ was optimized separately for each pair (see below, Hyperparameter optimization).

In a manner analogous to thinking of X_C_ as [ X_R_ X_L_], we can separate the retinal and LGN filters learned by the model as θ_C_ = [ θ_R_, θ_L_] (ignoring the additive offset term). For clarity, throughout this paper, when we refer to θ_R_ (or θ_L_), we are referring to θ_R_ transformed back into the time-domain:

(14) $$\theta_{\text{R}}=B_{\text{R}}\theta^{*}{}_{\text{R}}$$

(15) $$B_{\text{R}}=[b_{\text{R},\text{1}}\left(t\right)b_{\text{R},\text{2}}\left(t\right)...b_{\text{R},\text{k}}\left(t\right)],$$

where $b_{\text{R},\text{k}}\left(t\right)$ is the k-th basis for X_R_ (as above) and θ_R_ are the coefficients on X_R_ learned by the model.

The analogous set of relations apply to θ_L_, B_L_, etc.

##### Optimization error

For RH models, in which data used for fitting were represented in the standard temporal basis, the standard error (SE) of the estimate for each parameter was computed from the Hessian of the log-likelihood function ( ${▽}^{\text{2}}L(\theta_{\text{ML}})$) at the maximum likelihood estimate ( θ_ML_) following standard practice (Paninski, 2004; Truccolo et al., 2005; Paninski et al., 2007; Babadi et al., 2010):

(16) $$stderr\left(\theta_{\text{ML}}\right)=\text{diag}{\left({\left[\nabla^{2}L\left(\theta_{\text{ML}}\right)\right]}^{-1}\right)}^{\frac{1}{2}}.$$

The SE of parameter estimates for models fit to data represented in the raised-cosine basis are omitted from visualizations as the SE cannot be validly transformed back into the time-domain as the parameter estimates can.

#### Hyperparameter optimization

Hyperparameter values that could be chosen based on the literature or reasonable assumptions (in cases where a hyperparameter has little impact on the model overall) were fixed for all pairs at the values specified below. For quantifying model performance, all other hyperparameters were chosen for each pair from a predefined set based on which value yielded the highest cross-validated I_Bernoulli_ in a nested cross-validation procedure. On each fold of the main cross-validation loop the training-set (consisting of 90% of the total data from a pair) was further partitioned into subtraining and subtesting sets (again a 90–10 split); the combination of model parameters and hyperparameters that yielded the highest cross-validated I_Bernoulli_ on the subtesting set across subfolds were then used to quantify the model’s performance on the main testing-set.

##### ISI-efficacy model

The maximum ISI for which the ISI-efficacy model would predict a value other than the mean was chosen for each pair from a set of eight logarithmically spaced values (base 10) between 0.03 and 0.5 s. The SD of the Gaussian kernel used to smooth ISI-efficacy functions was chosen from a set of seven logarithmically spaced values (base 10) between 0.002 and 0.03 s and the value 0 (i.e., no smoothing).

##### RH model

The RH model contains two hyperparameters: the temporal span, which is the length of the time window preceding each target retinal spike that is used to train the model, and the prior weighting term, η, that controls the magnitude of the smoothness constraint (see above, RH models). The temporal span was chosen from a set of eight logarithmically spaced values (base 10) between 0.03 and 0.5 s (rounded to the nearest millisecond). The prior weighting term was chosen from a set of five logarithmically spaced values (base 2) between 4 and 4096.

##### CH model

As the CH model is an augmented version of the RH model, the temporal span of the retinal component of each pair’s CH model was fixed at the value derived from RH model fitting. Thus, seven hyperparameters remained: the temporal span of the LGN component, the number of basis vectors (in the raised-cosine basis; see above, CH models) used to represent each component (one hyperparameter per component), the weight given to the l^2^ penalty for each component, and the linearity of the basis vector spacing, Ψ (see above, CH models; again one per component). The temporal span of the LGN component was chosen from a set of eight logarithmically spaced values (base 10) between 0.04 and 0.6 s (rounded to the nearest millisecond). The number of basis vectors for retinal components was fixed at 16 for all pairs. For LGN components the number of basis vectors was chosen from the set {8, 12, 18, 24, 32}. The weight of the l2 penalty was chosen from a set of five logarithmically spaced values (base 2) between 0.125 and 8.0. The linearity of basis vector spacing, Ψ, was fixed at 10 and 8 for retinal and LGN components, respectively. The six-dimensional grid defined by the specified sets of values for the six nonfixed hyperparmeters was searched exhaustively.

#### Filter visualization

For visualizing and analyzing temporal profiles of the filters learned by the models, data from all pairs were fit with a fixed set of hyperparameters: temporal span for both RH and CH components was fixed at 200 ms, and the number of basis vectors in RH (CH) components was fixed at 16 (24). As they more directly affect the shape of the learned filters, prior weighting terms were chosen individually for each pair using 10-fold cross-validation from the same range specified above, in Hyperparameter optimization. When displaying averaged filters for a population or condition filters for each pair were scaled to have unit norm before averaging and, unless specified otherwise, error shading reflects the 95% confidence interval (CI) of the mean (see below, Statistics).

#### Burst spike definition

Geniculate bursts were identified by the criteria established by (Lu et al., 1992): a geniculate burst must be preceded by at least 100 ms of quiescence and contain two or more spikes each separated by no more than 4 ms. The relaxed definition reduced the quiescence duration to 50 ms and increased the maximum ISI to 6 ms (Fig. 4*C*,*D*). Noncardinal burst spikes were defined as all spikes that were part of an identified burst, except the first or “cardinal” spike of each burst.

#### Classification of retinal spikes by activity level

In order to assess how the level of activity of the early visual network might alter the integration dynamics of LGN cells we partitioned all retinal spikes from a given pair into four quartiles based on the LGN spike count in a 100-ms window preceding each retinal spike. RH models were then fit separately to data from each quartile. Differences between filters learned from data from distinct quartiles were quantified by taking the integral of the absolute difference between the two filters: $\int |\theta_{\text{N}}-\theta_{\text{M}}|.$ Where θ_N_ and θ_M_ are the filters learned the Nth and Mth quartiles, respectively. We refer to this metric as the “absolute difference” metric.

#### Simulating GLMs

Given a retinal spike train and a set of learned filter coefficients θ, a GLM can be used to simulate the relay status of the retinal spike train by constructing a predictor matrix X from the retinal spike train as described above (see above, RH model), multiplying X by the learned coefficients and passing the result through the logistic function (*X*θ) to attain the predicted relay probability for each retinal spike. Relay status y can then be simulated by drawing a random number for each retinal spike from a uniform distribution on (0,1); if the random number is less than the predicted probability for a given retinal spike the spike is considered relayed (this is equivalent to flipping a coin whose probability of heads, or in this case “relayed,” is given by the predicted relay probability). A GLM can then be fit to the retinal spike train and simulated relay status just as is done for real data (see above, RH model). Because of the stochastic nature of simulating relay status, for all simulations presented here the final two steps (simulate relay status and fit GLM) are repeated 50 times for each pair and the resulting coefficients are then averaged.

### Statistics

Unless otherwise noted in the text, data are reported as the median (or paired median difference) and the median absolute deviation (MAD) defined as: median(∣ x − median( x)∣) (Table 1). CIs are derived from bootstrap estimation with 5000 re-samples, and are bias corrected and accelerated (Efron, 1987) using the Bootstrap.jl software package (Gehring et al., 2021). For the awake dataset, the small sample size prevents the valid use of bootstrap-derived CIs; thus, we report the range of values ([min, max]) instead. For model comparisons, *p*-values are calculated from paired samples permutation tests with 5000 re-samples, where the permutation is performed within pair. For example, if comparing model A to model B, on each iteration the model performance values for each pair are randomly reassigned (i.e., swapped or not between A and B with probability 0.5) and the resulting paired median difference is calculated. After 5000 iterations the observed paired median difference is compared with the permuted differences distribution to yield a *p*-value. Computed *p*-values are then corrected so that they cannot be exactly zero (which would otherwise be possible given the discrete nature of the permuted differences distribution) using the method proposed in Phipson and Smyth (2010).

**Table 1:** Statistical table of results

	Dataset	Metric	Conditions	Paired median difference	MAD	95% CI	*p*-value
Figure 6							
a	Binary noise (*N* = 40)	I_Bernoulli_	RH–ISI	0.002 bits/spike	0.003	[0.000, 0.003]	0.0092
b	Binary noise (*N* = 40)	I_Bernoulli_	CH–RH	0.009 bits/spike	0.008	[0.004, 0.015]	0.0002
c	Binary noise (*N* = 40)	I_Bernoulli_	CH–ISI	0.004 bits/spike	0.004	[0.003, 0.009]	0.0002
Figure 7							
d	Gratings (*N* = 33)	I_Bernoulli_	RH–ISI	0.030 bits/spike	0.020	[0.012, 0.047]	0.0002
e	Gratings (*N* = 33)	I_Bernoulli_	CH–ISI	0.049 bits/spike	0.033	[0.032, 0.080]	0.0002
f	Gratings (*N* = 33)	I_Bernoulli_	CH–RH	0.020 bits/spike	0.014	[0.006, 0.027]	0.0002
Figure 8							
g	Gratings (*N* = 33)	I_Bernoulli_	Q4–Q1	−0.005 bits/spike	0.041	[−0.047, 0.003]	0.353
h	Binary noise (*N* = 39)	I_Bernoulli_	Q4–Q1	0.001 bits/spike	0.007	[−0.001, 0.004]	0.396
Figure 9							
i	Anesthetized (*N* = 27)	Absolute difference	Gratings–noise 100 ms	0.031	0.016	[0.018, 0.039]	0.0002
j	Anesthetized (*N* = 27)	Absolute difference	Gratings–noise 30 ms	0.008	0.007	[0.002, 0.012]	0.0004

CIs are derived from 5000 bootstrap resamples and are bias corrected and accelerated; *p*-values are derived from paired-permutation tests with 5000 permutations. For details, see Materials and Methods.

## Results

**Figure 1.**
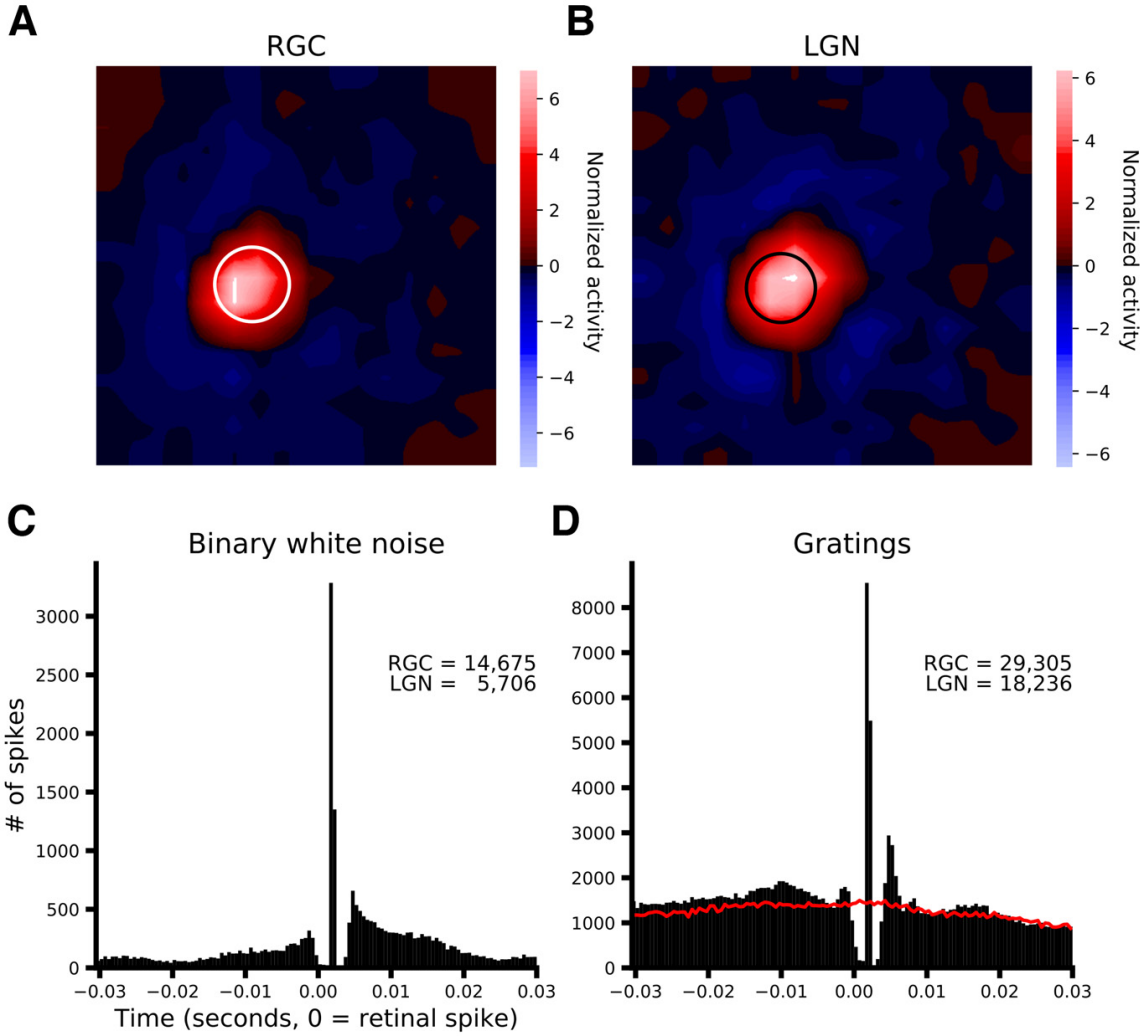
Data from an example pair (pair 214). ***A***, ***B***, RF maps derived from reverse correlation between recorded spike trains and binary white noise stimulus. Red (blue) denotes regions of the RF that were excited by brighter (darker) pixels. White (black) circle in ***A*** (***B***) is the 1 SD contour of a circular Gaussian fit to RF of the LGN cell (RGC) overlayed on the RGC (LGN) RF to illustrate the high degree of spatial overlap. ***C***, ***D***, Cross-correlation between RGC and LGN spike trains for binary white noise (***C***) and drifting sinewave grating (***D***) stimuli. The inset text indicates the number of spikes recorded from each of the two neurons (***C***, 14,675 retinal spikes, 5706 LGN spikes; ***D***, 29,305 retinal spikes, 18 236 LGN spikes). The red line in ***D*** shows the correlation because of the stimulus that is attained if the spike train of the RGC is shifted in time by one stimulus cycle.

To investigate the factors that contribute to how the LGN filters retinal spike trains, we analyzed data from 45 monosynaptically connected RGC-LGN cell pairs from anesthetized cats and 8 pairs from awake cats. For the recordings under anesthesia, neurons were stimulated with binary white noise (*n* = 40) and/or drifting sinewave gratings (*n* = 33) and connectivity was assessed by cross-correlation of the spike times from the two simultaneously recorded neurons. Figure 1 shows data from an example pair. The top row (Fig. 1*A*,*B*) shows RF maps of the RGC (left) and LGN neuron (right) derived from the spike-triggered average of the binary white-noise frames. The one SD contour of a circularly symmetric Gaussian fit to the LGN (RGC) RF is overlayed in white (black) on the RGC (LGN) RF, demonstrating the high degree of spatial overlap between the two RFs. The bottom row (Fig. 1*C*,*D*) shows the cross-correlograms, LGN spike times relative to each RGC spike, for the two stimulus conditions for this pair. Using a monosynaptic latency derived from the time lag at which the cross-correlogram peaks, we identified each retinal spike as being relayed (i.e., evoked a spike in its LGN partner) or not, and each LGN spike as being triggered (i.e., was evoked by a RGC spike) or not. Retinal efficacy, the proportion of retinal spikes that were relayed (see Materials and Methods, Identification of monosynaptically connected pairs and relayed RGC spikes), for this example pair during binary white noise (drifting grating stimuli) was 0.316 (0.473); retinal contribution, the proportion of LGN spikes that were triggered, was 0.812 (0.760). Across the population, for binary white noise data median retinal efficacy was 0.097 (MAD 0.070, 95% CI [0.054, 0.173]) and median retinal contribution was 0.247 (MAD 0.156, 95% CI [0.136, 0.394]); for drifting grating data median retinal efficacy was 0.161 (MAD 0.098, 95% CI [0.088, 0.229]) and median retinal contribution was 0.347 (MAD 0.197, 95% CI [0.205, 0.490]).

Additionally, we analyzed data from a smaller set of eight RGC-LGN cell pairs from awake cats in which the spike train of the connected RGC was inferred from the presence of S-potentials that could be isolated, along with the LGN cell’s spikes, from the extracellular voltage trace recorded within the LGN (Weyand, 2007). Across the population, median retinal efficacy was 0.519 (MAD 0.133, range [0.154, 0.724]) and median retinal contribution was 0.935 (MAD 0.037, range [0.604, 0.997]).

Importantly, these data confirm the well documented finding that not every retinal spike is relayed by the LGN (Cleland et al., 1971; Kaplan and Shapley, 1984; Kaplan et al., 1987; Usrey et al., 1998; Sincich et al., 2007; Weyand, 2007, among others). Taken together with the generally accepted notion that every nonburst relay cell spike is triggered by the retina (Kaplan and Shapley, 1984; Sincich et al., 2007; Weyand, 2007), this finding suggests that the primary role of LGN relay cells is to edit the incoming retinal spike train by selective deletion. Thus, we sought to investigate the factors that determine which retinal spikes are relayed and which are not, what we term “relay status.” Given this goal, we consider models of retinogeniculate transmission that focus specifically on predicting the relay status of retinal spikes rather than trying to predict the LGN spike train directly (i.e., we do not attempt to predict LGN spikes that were not triggered by the recorded RGC).

### ISI-efficacy model

**Figure 2.**
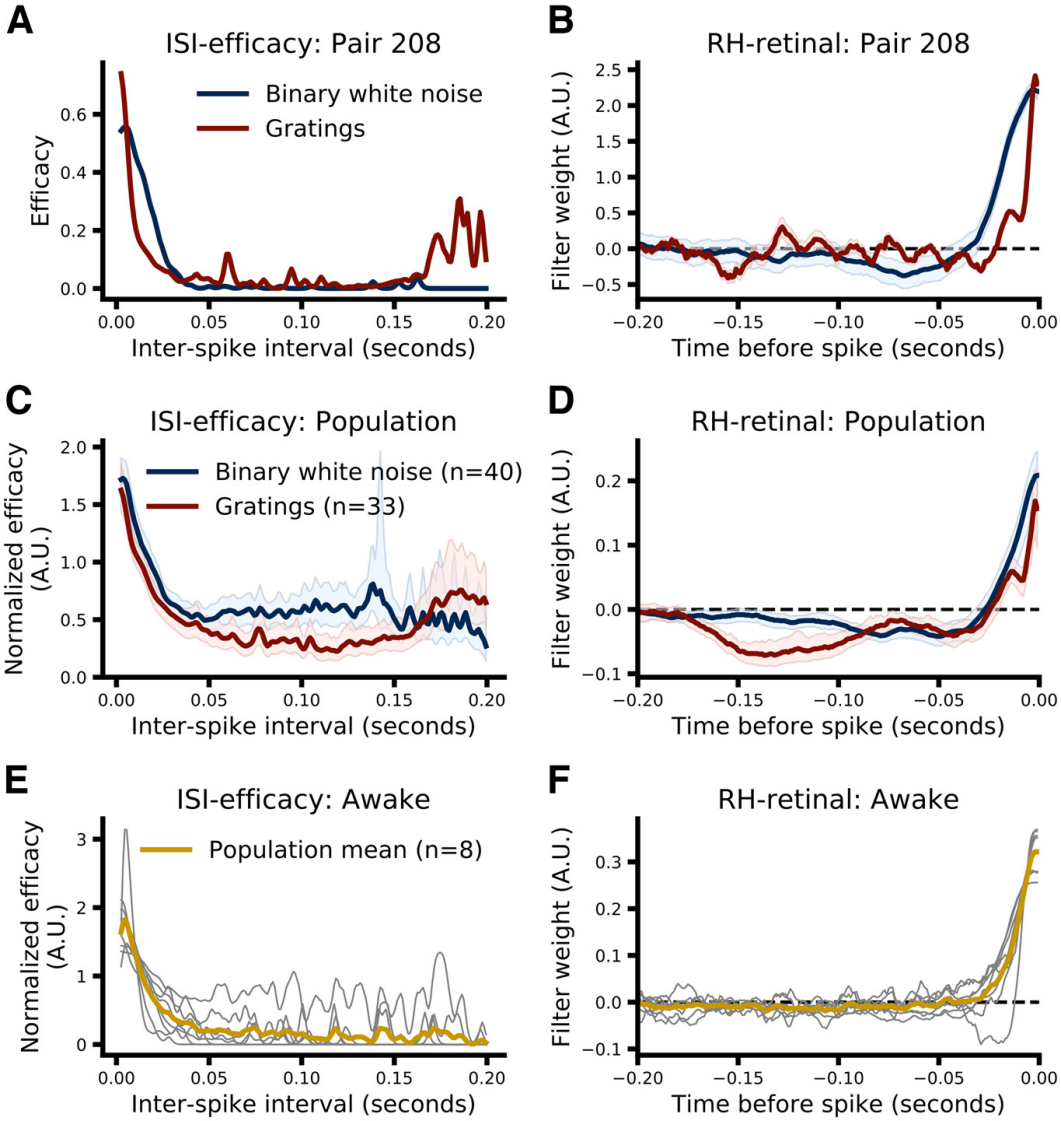
Comparison of ISI-efficacy (left column) and RH (right column) models. ***A***, Relationship between retinal ISI and retinal efficacy for binary white noise (blue) and drifting grating data (red) for pair 208. ***B***, Retinal filters learned by the RH model fit to binary white noise (blue) and drifting grating (red) data from pair 208. Shading indicates ±1 SE of the optimization (see Materials and Methods). The time base for GLM filters is always relative to the retinal spike about which a prediction (relayed or nonrelayed) is being made (i.e., the “target spike”). ***C***, Normalized ISI-efficacy relation averaged across the population. Efficacies for each pair were normalized to the mean efficacy across all ISIs for that pair before averaging. Shading represents the 95% CI across pairs from 5000 bootstrap resamples (see Materials and Methods, Statistics). ***D***, Same as ***B*** but showing the average filters across pairs. Filters fit to the data from each pair were scaled to have a unit norm before averaging. Shading represents the 95% CI across pairs. ***E***, Normalized ISI-efficacy relations for all eight pairs from the awake dataset (thin gray lines) and the population average (thick gold line). Normalization was performed as in ***C***. ***F***, Retinal filters learned by RH models fit to data from each pair in the awake dataset (thin gray lines) and the population average (thick gold line). Filters were scaled to have unit norm (as in ***D***) to aid visualization.

Previous work has clearly demonstrated that one of the primary factors that determines which retinal spikes are relayed is the elapsed time since the last retinal spike (i.e., retinal ISI; Usrey et al., 1998; Carandini et al., 2007; Sincich et al., 2007, 2009; Casti et al., 2008; Wang et al., 2010). This is often visualized by plotting retinal efficacy as a function of the preceding retinal ISI (Usrey et al., 1998; see above, ISI models). Figure 2, left column, shows the ISI-efficacy relation for an example pair of cells from the anesthetized dataset (A, pair ID 208), the population as a whole (C, anesthetized dataset), and the relations for each pair in the awake dataset (E), where the data from each pair in C and E were normalized to their mean before averaging. The ISI-efficacy functions follow the typical decay pattern (shorter ISIs in general show higher efficacies) that has been reported previously (Usrey et al., 1998; Weyand, 2007; Rathbun et al., 2010). Interestingly, the drifting grating data (Fig. 2*C*, red line) do show a slight increase in efficacy for ISIs >150 ms, potentially caused by the release from a slow acting suppressive influence such as synaptic depression. Implicitly, the ISI-efficacy relation is a simple model for predicting which retinal spikes were relayed based on the preceding retinal ISI (Wang et al., 2010), thus we formalized the model to quantitatively access its decoding performance. We used a 10-fold cross- validation procedure in which ISI-efficacy functions were constructed using 90% of retinal spikes (training set), and performance was assessed on the remaining 10% (test set) by looking up the expected efficacy of each spike in the test set from the training-set-derived ISI-efficacy function (see above, ISI models). This procedure was repeated ten times such that each retinal spike was included in the test set once and model performance was evaluated by the cross-validated, single-spike Bernoulli information (I_Bernoulli_) which quantifies how informative model predictions are about the relay status of test-set retinal spikes relative to a homogeneous model that always predicts the mean efficacy (see Materials and Methods, Assessing model performance). For binary, white-noise data, median I_Bernoulli_ was 0.019 bits/spike (MAD 0.018, 95% CI [0.004, 0.041]). For drifting grating data, median I_Bernoulli_ was 0.026 bits/spike (MAD 0.017, 95% CI [0.010, 0.030]). For the awake dataset, median I_Bernoulli_ was 0.177 bits/spike (MAD 0.085, range [0.075, 0.439]). This demonstrates that the ISI-efficacy model was able to predict the relay status of retinal spikes significantly better than the homogeneous model regardless of the stimulus condition or the state of the animal (anesthetized or awake).

### RH model

While retinal ISI is a strong predictor of relay status, its influence is a natural consequence of the temporal integration that occurs within relay cells. This fact suggests that the history dependence of relay probability is likely to extend beyond the most recent spike and might be better captured by considering all retinal spikes that occur within a given window of time. Thus, we sought to extend the ISI-efficacy model by using GLMs to predict the relay status of retinal spikes based on the patterns of retinal activity preceding each spike, what we call the retinal history (RH) model. Historically, GLMs have been used to predict the activity of visual neurons based on the changing pattern of a visual stimulus (Chichilnisky, 2001; Paninski, 2004; Truccolo et al., 2005; Pillow et al., 2008; Babadi et al., 2010); here, we instead use the pattern of activity recorded simultaneously from a monosynaptic input (see Generalized linear models). In brief, the GLM predicts the relay status of a retinal spike by convolving the pattern of recent activity with a learned temporal filter, the output of which is then passed through a static nonlinearity to produce a relay probability. Specifically, we use Bernoulli-Logistic GLMs (i.e., logistic regression) to model retinogeniculate transmission as a binary parsing (Wang et al., 2010) or coin-flip process where the probability of a positive outcome (relayed retinal spike) varies continuously over time as a function of the pattern of recent retinal spikes (see Materials and Methods, Retinal history models).

Figure 2, right column, shows the temporal filters learned from drifting grating (red) and binary, white-noise data (blue) for an example pair (Fig. 2*B*, pair ID 208), the population recorded under anesthesia (Fig. 2*D*), and the population recorded in the awake state (Fig. 2*F*), where filters from each pair were scaled to have unit norm before averaging in D and F. For visualization purposes, the time span preceding each retinal spike that the model could consider (temporal span) was set to 0.2 s for all pairs (see [Visualizing filters] and Materials and Methods, Hyperparameter optimization). Much like the ISI-efficacy functions, the temporal filters show relatively large positive values in the time window just before the target retinal spike (at *t* = 0), indicating that retinal spikes falling within this time window increase the likelihood that the target retinal spike will be relayed. Retinal spikes that occurred earlier relative to the target spike (>0.02–0.04 s) were less informative about relay status, as shown by the smaller magnitude of the filter values, and in general tended to slightly decrease the probability that the target retinal spike would be relayed (i.e., filter values slightly <0) for pairs recorded under anesthesia. Interestingly, the filters learned from drifting grating data tended to have larger negative values during earlier prespike time windows (>∼0.08–0.18 s prespike) and show a slight oscillation at ∼10 Hz, which is unlikely to be due solely to the periodic nature of the drifting grating, which had a temporal frequency of 4 Hz (see Materials and Methods, Electrophysiological recording and visual stimuli). As with the ISI-efficacy model, the performance of the RH model was assessed using a train-on-90% test-on-10%, 10-fold cross-validation procedure, in which overall performance was computed as the average I_Bernoulli_ across folds. For binary, white-noise data, median I_Bernoulli_ was 0.024 bits/spike (MAD 0.022, 95% CI [0.007, 0.042]). For drifting grating data, median I_Bernoulli_ was 0.068 bits/spike (MAD 0.047, 95% CI [0.036, 0.099]). For the awake dataset, I_Bernoulli_ was 0.154 bits/spike (MAD 0.072, range [0.061, 0.452]).

### CH model

Although the recorded RGC could account for the majority of LGN spikes in many cell pairs (i.e., a retinal contribution >0.5), a considerable number of LGN spikes could not be directly attributed to (i.e., were not triggered by) the recorded RGC. These nontriggered spikes likely represent the activity of other RGC inputs to the recorded relay cell (Usrey et al., 1999), and might provide a nonredundant source of information that could aid predictions about which RGC spikes were relayed and which were not. Thus, we built an augmented version of the RH model that included an additional filter that acted on the spiking history of the recorded LGN relay cell, what we call the combined history (CH) model. Two attributes of this additional filter are worth noting: (1) the activity of the LGN cell only contributes to the RH model by identifying which retinal spikes were relayed. Thus, for any pair with a retinal contribution less than one, the LGN activity may contribute additional information that the model can take advantage of, and (2) the LGN filter is aligned relative to the time of the target retinal spike just as the retinal filter is, so only LGN spikes that occurred before target retinal spike are included (see Materials and Methods, Combined history models). This construction is distinct from those commonly used to represent spike history effects in GLM models (Pillow et al., 2008; Babadi et al., 2010) and reflects our focus on predicting the relay status of retinal spikes and not the activity of the LGN cell per se. As a result, the LGN filter can capture some features of LGN activity, such as bursting in certain circumstances, but not others, such as a refractory period, which is not relevant for predicting retinal relay status.

**Figure 3.**
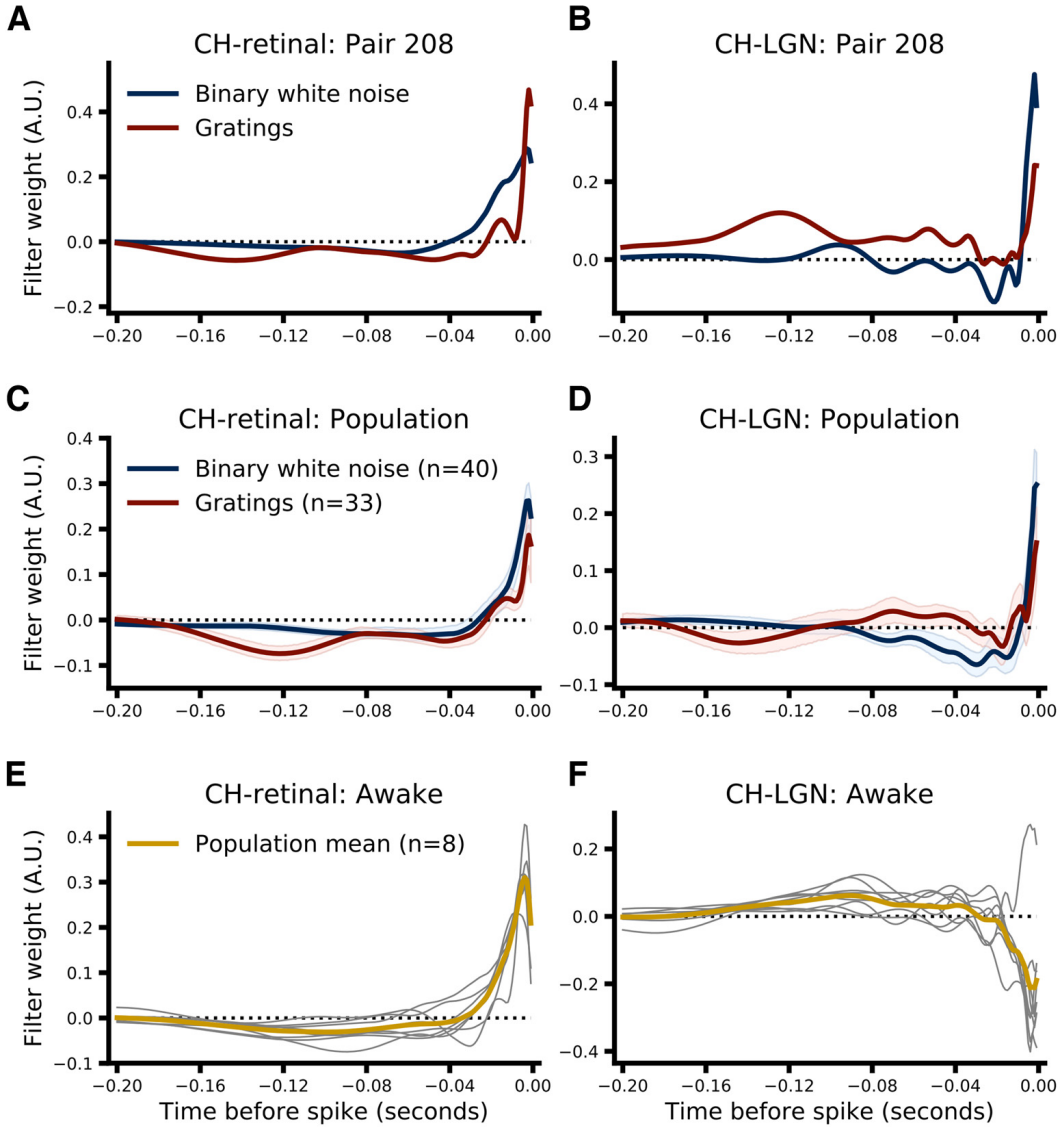
Summary of filters learned by the two-component, CH model. The left column shows the retinal filters, and the right column shows the LGN filters for example pairs and the population for each dataset. ***A***, Retinal filters learned by the CH model for binary white noise (blue) and drifting grating (red) data from pair 208. ***B***, Same as ***A*** but showing the LGN filters learned by the CH model. The time base for retinal and LGN filters is the same (0 is the time of the “target” retinal spike), but LGN filters operate on the prior activity of the LGN cell. ***C***, Same as ***A*** but for the population. Filters fit to the data from each pair were scaled to have a unit norm before averaging. Shading represents 95% CI across pairs. ***D***, Same as ***C*** but for LGN filters. ***E***, Same as ***C*** but showing retinal filters learned from the awake dataset (thin gray lines show filters from each pair, the thick gold line shows the mean across pairs). ***F***, Same as ***E*** but showing LGN filters.

**Figure 4.**
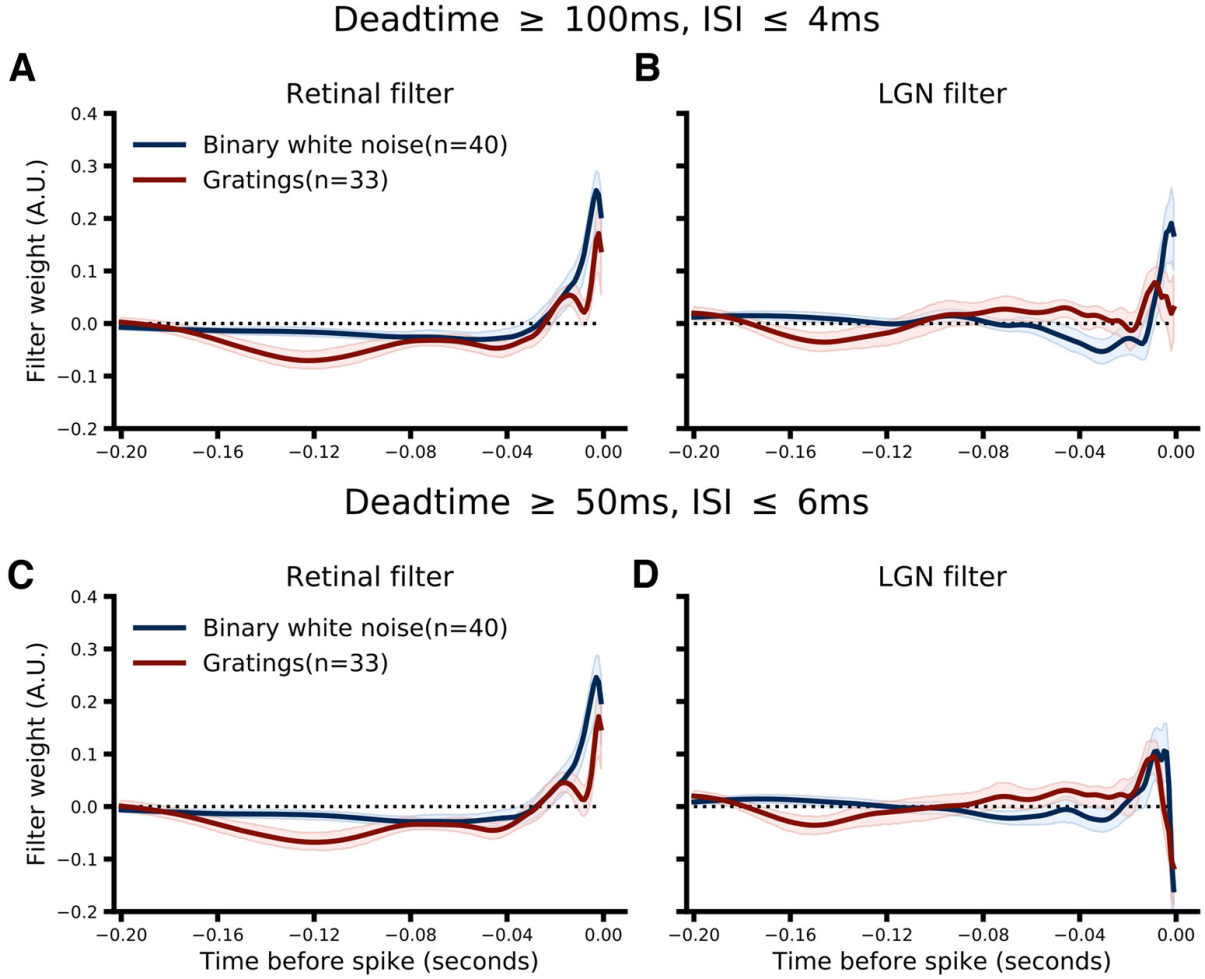
Retinal (***A***, ***C***) and LGN (***B***, ***D***) filters from the CH model fit to data where noncardinal burst spikes were first removed. The first row (***A***, ***B***) use the classic burst spike definition by Lu et al. (1992): a quiescent period ≥100 ms followed by two or spikes with ISIs ≤4 ms. The second row (***C***, ***D***) use a more relaxed criteria: a quiescent period ≥50 ms followed by two or more spikes with ISIs ≤6 ms.

Figure 3 shows the retinal (Fig. 3*A*) and LGN (Fig. 3*B*) filters for an example pair (pair ID 208) and the population as a whole (Fig. 3*C*,*D*, filters from each pair were scaled to have unit norm before averaging). For visualization purposes, the temporal span of both retinal and LGN filters was set to 0.2 s for all pairs (see Materials and Methods, Filter visualization). Two aspects of the filters learned by the CH model are worth noting. First, the shape of the retinal filters are nearly identical to the shape of the retinal filters learned by the RH model as expected (compare Figs. 2*D* and 3*C*), despite using far fewer parameters (see Materials and Methods, Combined history models), suggesting that the addition of the LGN filter has not fundamentally changed how the model is weighting retinal spikes in making predictions. Second, much like the retinal filters, the LGN filters show a strong positive component immediately preceding the target spike that rapidly declines (∼−0.015 s) followed by a lower amplitude negative component that decays to near zero fairly quickly for drifting grating data (∼0.04 s; Fig. 3*C*, red) and more slowly for binary white noise data (∼0.1 s; Fig. 3*C*, blue). The strong, positive weights assigned by the model to the time window immediately preceding the target spike suggests that retinal spikes that follow LGN spikes at very short latencies are more likely to be relayed. This pattern of LGN-RGC-LGN spiking is expected to be particularly likely when a retinal spike arrives during a geniculate burst (Llinás and Jahnsen, 1982; Huguenard and McCormick, 1992; Alitto et al., 2019b). To test whether this filter component was in fact due to LGN bursting, we repeated the CH model fitting procedure after removing all noncardinal burst spikes (i.e., removing all spikes that comprise a burst except the first spike; see Materials and Methods, Burst spike definition). Interestingly, while the resulting filters do show a strongly attenuated early positive component for the drifting grating data, removing all noncardinal burst spikes only minimally altered the LGN filters learned from binary white noise data (Fig. 4*B*). However, relaxing the definition of bursts somewhat to include more high-frequency events reduced the early positive component for binary white noise data (Fig. 4*D*), suggesting that the early positive component of LGN filters may reflect both burst as well as high-frequency, nonburst events (Alitto et al., 2019b).

The retinal filters learned by the CH model from the awake data (Fig. 3*E*) closely resembled those learned from the anesthetized data, as expected from the RH model results (Fig. 2*D*,*F*). However, the LGN filters learned from the awake data show a very different pattern. Instead of the short latency, positive component that appears to be due in large part to LGN bursting (see above), the LGN filters for seven of the eight pairs of the awake dataset show a clear, negative component over the same time span (∼−0.03–0.0 s preceding the target spike). Two aspects of this observation are worth noting. First, LGN cells in the awake dataset produced very few bursts. Averaged across pairs only 0.235% (median 0%, range [0.0, 1.52]) of LGN spikes were part of bursts, with five of the eight producing no bursts at all by the accepted definition (Lu et al., 1992; see Materials and Methods, Burst spike definition). In comparison, across pairs from the anesthetized datasets the median percentage of spikes that were part of bursts was much higher: 14.203% (MAD 9.698, 95% CI [9.223, 17.496]) for the binary white noise dataset, and 18.264% (MAD 14.805, 95% CI [9.792, 27.777]) for the drifting grating dataset. Thus, the lack of the positive component seen in the anesthetized data are expected. Second, the negative component of the LGN filters suggests that some form of gain control or normalization is occurring. This follows from the construction of the model, negative LGN filter weights over some time interval indicate that LGN spikes that occur during that interval will push the model toward predicting that the target spike will not be relayed, thus lowering the activity of the LGN cell itself and producing a gain control or normalization-like effect (i.e., the same retinal input produces a smaller magnitude response when the LGN has just been active compared with when it has just been quiescent; Shapley and Enroth-Cugell, 1984).

As with previously discussed models, the performance of the CH model was assessed using 10-fold cross-validation procedure. For binary white noise data median I_Bernoulli_ across pairs was 0.033 bits/spike (MAD 0.030, 95% CI [0.015, 0.064]), and for drifting grating data median I_Bernoulli_ was 0.073 bits/spike (MAD 0.051, 95% CI [0.051, 0.134]). For the awake dataset, median I_Bernoulli_ was 0.263 bits/spike (MAD 0.083, range [0.086, 0.489]). Consistent with the idea that CH-LGN filters may be capturing the effect of LGN bursts in the anesthetized dataset, we observed that the gain in performance of CH models compared with RH models across pairs was fairly well correlated with the “burstiness” of the LGN cell of each pair. The Spearman’s correlation between I_Bernoulli_ difference (CH – RH) and percentage of LGN spikes that were part of bursts (not including cardinal spikes, see Materials and Methods, Burst spike definition) was 0.500 (95% CI [0.182, 0.726], *p *<* *0.01) for binary white noise data, and 0.286 (95% CI [−0.087, 0.569], *p *≈* *0.1) for drifting grating data (Extended Data Fig. 7-1*C*).

10.1523/ENEURO.0088-22.2022.f7-1 Extended Data Figure 7-1 Correlates of model performance. ***A***, Left, Residual Spearman’s correlation betweenI_Bernoulli_ from RH models and retinal contribution where the effect of retinal efficacy on each variable has been removed prior to the analysis. Right, Estimation of correlation coefficient using 5000 bootstrap resamples. Black dots denote point estimates, vertical black lines denote 95% CI, and filled distributions summarize the results of the resampling. ***B***, Left, Spearman’s correlation between model performance improvement (ΔI_Bernoulli_) between CH and RH models and retinal contribution. As retinal efficacy is not correlated with ΔI_Bernoulli_ regular Spearman’s correlation was used. Right, estimation analysis for correlation shown at left. ***C***, Left, Spearman’s correlation between ΔI_Bernoulli_ and the percent of LGN spikes that were part of identified bursts (using the traditional criteria by Lu et al., 1992) excluding the cardinal spike of each burst. Right, Estimation analysis for correlation shown at left. Download Figure 7-1, TIF file.

### Model comparison

**Figure 5.**
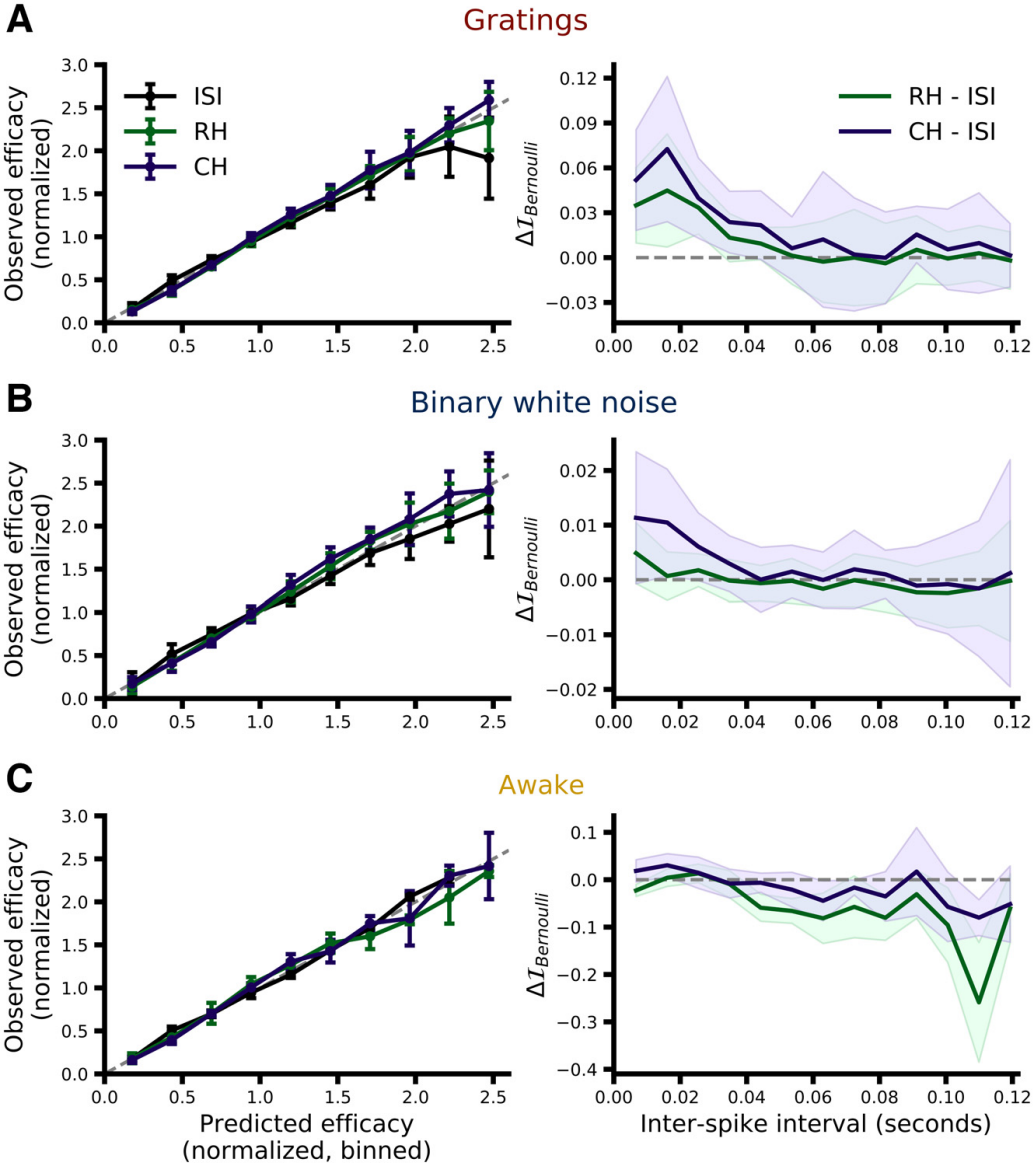
Qualitative comparison of model performance. ***A***, Left, The predicted efficacies from each model were used to group retinal spikes into bins, and the observed efficacy for each group (median across pairs) is plotted against the corresponding bin label (error bars represent the MAD across pairs). Both predicted and observed efficacies from each pair were normalized by the mean efficacy of that pair before calculating the median and MAD. ***A***, Right, The performance ( I_Bernoulli_) of the GLMs relative to the ISI-efficacy model is shown as a function if ISI. Lines show the median performance difference across pairs; shading represents the MAD. ***B***, ***C***, Same as ***A*** but for the binary white noise (***B***) and awake (***C***) datasets.

In order to illustrate how well each model performed relative to the others we first examined how well the model-predicted efficacies correlated with the observed efficacies. To do this we grouped the retinal spikes from each pair according to their predicted efficacy (normalized by the mean efficacy of that pair), calculated the observed efficacy for each group (also normalized within-pair by that pair’s mean efficacy), and then plotted the normalized, observed efficacy against the normalized, predicted efficacy. Efficacies, both predicted and observed, for each pair were normalized by the observed mean efficacy of that pair (across all spikes) to account for the large difference in efficacy across pairs as is typically done (Alitto et al., 2019a,b). In such a framework, a well performing model will produce a “unity” line with a slope of one and y-intercept of zero (i.e., predicted efficacy and observed efficacy match). Figure 5, left column, shows, for each dataset, the median relationship between observed and predicted efficacy for each model (error bars represent the MAD across pairs). While all models appear to perform quite well within this framework, there is a systematic trend for the ISI-efficacy model to perform worse for the spikes that it predicts to have the highest efficacy within the drifting grating and binary white noise datasets. Given that the highest efficacy spikes should follow short ISIs (Fig. 2), this suggests that the ISI-efficacy model may be performing worse than the GLMs specifically for short ISI spikes. Consistent with this suggestion, Figure 5, right column, shows that the difference in I_Bernoulli_ between the GLM and ISI-efficacy models is most pronounced for retinal spikes with the shortest ISIs within the drifting grating and binary white noise datasets. Interestingly, within this comparison framework the ISI model appears to perform as well as the GLMs on the awake dataset.

**Figure 6.**
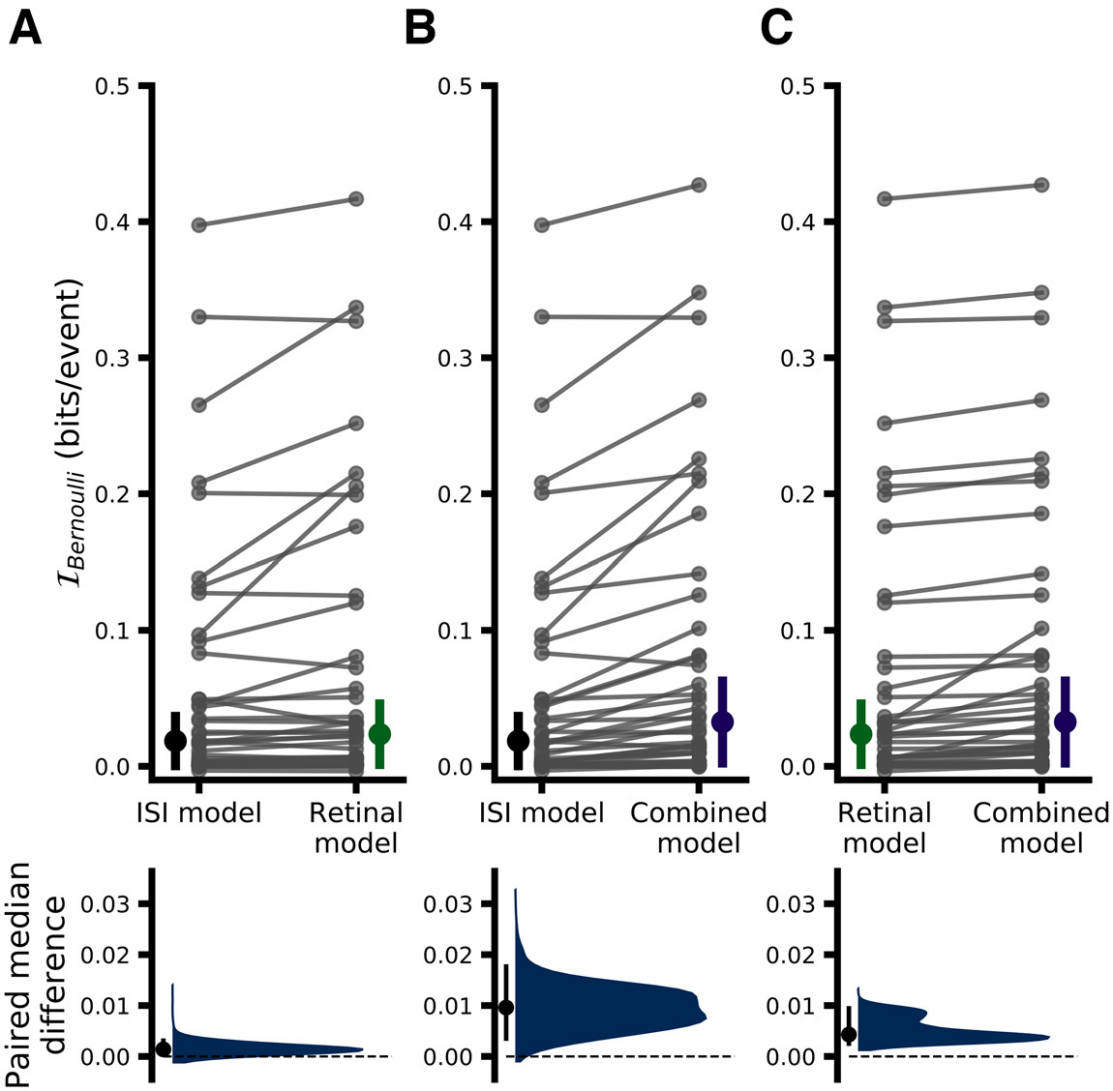
Performance comparison of all models for binary white noise data. ***A***, Upper, Comparison of ISI-efficacy and RH models. Each dot indicates the meanI_Bernoulli_ for a given pair and model; lines connect data belonging to the same pair across models (thus the slope of the lines depicts the change inI_Bernoulli_). The height of the vertical, colored bars indicates the MAD ofI_Bernoulli_ across pairs for a given model, with the filled circle indicating the median value. ***A***, Lower, Estimated paired median differenceI_Bernoulli_ between ISI-efficacy and RH models. The black dot indicates the observed paired median difference and the vertical black line indicates the 95% CI of the bootstrap distribution (5000 samples) shown in blue. ***B***, Same as ***A*** but comparing ISI-efficacy and CH model performance. ***C***, Same as ***A*** but comparing RH and CH model performance.

**Figure 7.**
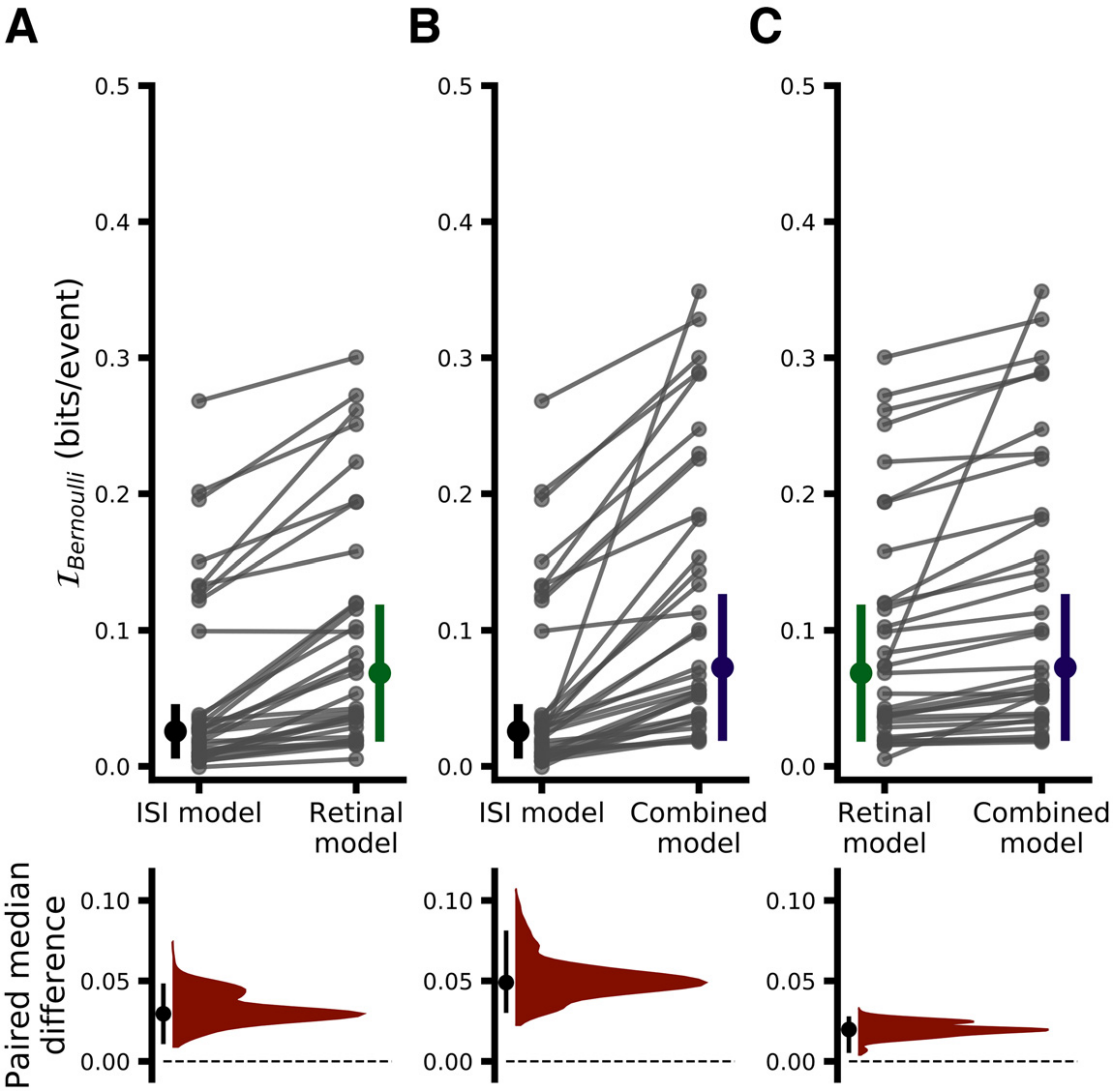
Performance comparison of all models for drifting grating data. All conventions exactly follow those from Figure 6. Correlates of model performance are shown in Extended Data Figure 7-1. Model performance for the awake dataset is shown in Extended Data Figure 7-2.

While Figure 5 provides a helpful overview of model performance, given the present context the most rigorous way to assess the performance of the models presented here is using I_Bernoulli_, the cross-validated single-spike Bernoulli information, which quantifies the accuracy of model predictions on a spike-by-spike basis. Figures 6 and 7 summarize the results of a direct model comparison analysis for the binary white noise and drifting grating data, respectively, in which all hyperparameters for all models were optimized individually for each pair (see Materials and Methods, Hyperparameter optimization). The top row of each figure shows the cross-validated I_Bernoulli_ for each pair and each model, where points corresponding to the same pair are connected, and the bottom row shows a bootstrap estimation of the paired median difference in I_Bernoulli_ between models (see Materials and Methods, Statistics). For the binary white noise data (Fig. 6), the paired median difference between ISI-efficacy and RH models was 0.002 bits/spike (MAD 0.003 95% CI [0.000, 0.003], *p *≈* *0.0092)^a^, between ISI-efficacy and CH models was 0.009 bits/spike (MAD 0.008 95% CI [0.004, 0.015], *p *≈* *0.0002)^b^, and between RH and CH models was 0.004 bits/spike (MAD 0.004 95% CI [0.003, 0.009], *p *≈* *0.0002)^c^. For the drifting grating data (Fig. 7), the paired median difference between ISI-efficacy and RH models was 0.030 bits/spike (MAD 0.020 95% CI [0.012, 0.047], *p *≈* *0.0002)^d^, between ISI-efficacy and CH models was 0.049 bits/spike (MAD 0.033 95% CI [0.032, 0.080], *p *≈ 0.0002)^e^, between RH and CH models was 0.020 bits/spike (MAD 0.014 95% CI [0.006, 0.027], *p *≈* *0.0002)^f^.

10.1523/ENEURO.0088-22.2022.f7-2 Extended Data Figure 7-2 Model comparison and activity level analysis for awake data. ***A***, Model performance (mean I_Bernoulli_ across folds) for each model and pair (grey points) where grey lines connect points that correspond to the same pair. Large, solid color circles indicate the median, and solid-color vertical lines show the MAD, across pairs for a given model (black: ISI model, green: RH model, purple: CH model). ***B***, Retinal filters learned by RH models from low (green) and high (purple) activity datasets (similar to Fig. 6 but using a median split to assign each retinal spike to a dataset). Filters from individual pairs are show in less saturated, thin lines while thick saturated lines indicate the mean across pairs (all filters are scaled to have unit norm to aid visualization). Inset axis highlights the boxed region corresponding to the 30 ms immediately preceding each “target spike” (at *t* = 0). The red arrows indicate the filters learned from pair 200001250, which is the only pair of the awake dataset that was stimulated with gratings during recording. Download Figure 7-2, TIF file.

While the small size of the awake dataset precludes a statistical comparison of model performance, a qualitative assessment shows largely the same pattern as seen in the anesthetized data. Extended Data Figure 7-2*A* illustrates the pairwise difference in model performance between the three models (ISI, RH and CH) which suggests that although no difference between the performance of the ISI and RH models is evident, the inclusion of the LGN filter in the CH model may substantially improve performance (median pairwise difference in I_Bernoulli_ between RH and CH models was 0.058 bits/spike, range [−0.004, 0.170]).

Overall, while RH models do show significantly better performance than ISI-efficacy models, and CH models significantly outperform RH models, the magnitude of the performance gain is rather modest, suggesting that, overall, retinal ISI is the dominant factor in determining which retinal spikes are relayed. However, while both stimulus conditions showed this trend, the magnitude of the performance gain associated with RH and CH models over the ISI-efficacy model was substantially larger when pairs were stimulated with drifting gratings, suggesting that some subtler aspects of LGN integration may differ between the two stimulus conditions (Figs. 2*D*, 3*C*; Usrey et al., 1998).

### Integration dynamics depend on firing rate

**Figure 8.**
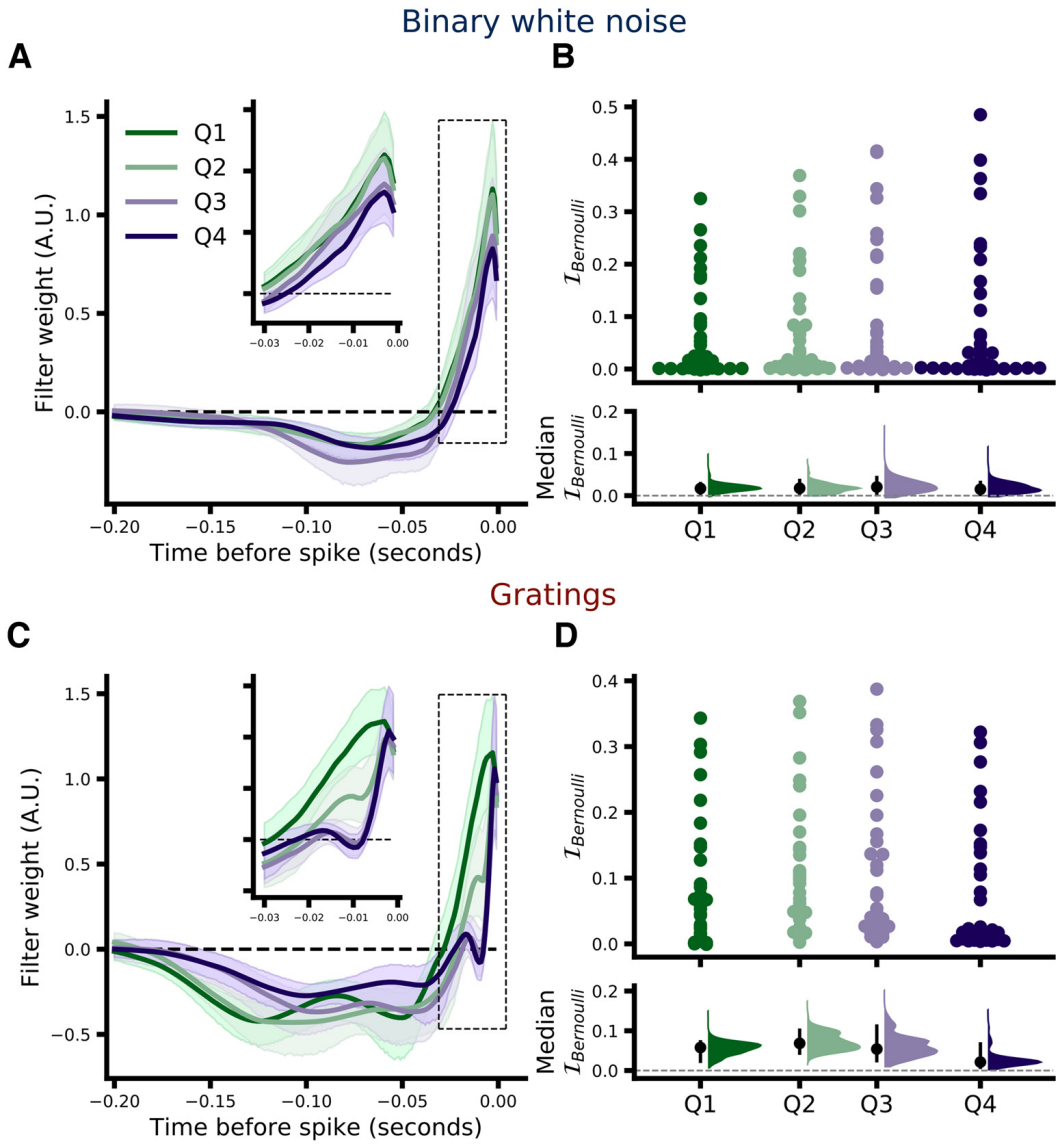
Comparison of RH models fit separately to subsets (quartiles) of the data grouped by LGN activity level. ***A***, Average retinal filters from RH models fit to each quartile of the binary white noise dataset from low (Q1, green) to high (Q4, purple) based on the activity level of the LGN neuron within a 100-ms period directly preceding the target retinal spike att = 0. Shading represents 95% CI across *N* = 38 pairs. ***B***, Upper, Comparison of model performance (I_Bernoulli_) across all activity subsets. Each dot represents the model performance for a single pair (the spread along the *x*-axis is to aid visualization). ***B***, Lower, Bootstrap estimation of median model performance for each subset. Black dots indicate the median across pairs and black vertical lines indicate the 95% CI of the bootstrap distribution (shown in color, 5000 samples). ***C***, ***D***, Same as ***A***, ***B***, but for the drifting gratings dataset (*N* = 33). Results from a control analysis wherein relay status was simulated via GLMs is shown in Extended Data Figure 8-1 (see main text for details). Results of changing the spike quartile classification window are shown in Extended Data Figure 8-2.

One potential drawback of using GLMs in the present context is that by fitting a single set of filters to all spikes (or a random subset), we are asking the fitting algorithm to find what amounts to the average integration behavior of relay cells during the recording period. The analysis is, by design, insensitive to any changes in relay cell integration that may occur within a stimulus condition. While this implicit assumption of stationarity may be largely valid for the binary white noise stimulus, it may not hold during drifting grating stimulation because of the high degree of spatial and temporal correlations present in drifting gratings, which are of course absent from the binary white noise. The strong correlations present in drifting gratings may result in larger fluctuations in activity for both the RGC-LGN cell pair being recorded as well as the wider network (including, e.g., the thalamic reticular nucleus, V1, etc.) and thus may alter LGN integration dynamics in a more significant manner. Consistent with this idea, we observed higher RGC firing rate variability during drifting grating stimulation in the 200-ms period immediately preceding each retinal spike (the same time period that the model could consider): median pairwise difference in firing rate SD (gratings minus binary white noise) was 3.549 spikes/s (MAD 4.581, 95% CI [0.152, 5.844]; median 16.768 and 11.587 spikes/s for gratings and binary white noise, respectively). The models presented thus far are not sensitive to these potential within-condition changes, as each model is fit to all spikes (or a randomly selected subset) from a single stimulus condition. Thus, we sought to investigate specifically whether LGN integration dynamics might differ based on the level of activity by assigning each retinal spike to one of four “quartile” subsets (Q1–Q4) of the data based on the quartile into which the LGN spike count in a 100-ms window preceding each retinal spike fell (see Materials and Methods, Classification of retinal spikes by activity level). We then fit separate GLMs to the data from each quartile for each stimulus type. For this analysis we consider only RH models, as the quartile partitioning results in too few LGN spikes in the lowest activity quartile to reliably fit CH models. Additionally, the binary white noise data from one pair (pair ID 102) did not contain enough spikes to reliably fit RH models for each quartile and was excluded from activity level analyses. Figure 8 shows the filters learned by the model for each activity level and stimulus condition averaged across pairs (filters from each pair were scaled to have unit norm before averaging) where the shaded regions represent the 95% CI across pairs (see Materials and Methods, Filter visualization). For binary white noise (Fig. 8*A*), there is an apparent trend toward a small difference between ∼40 and 120 ms preceding the target spike (at time = 0) such that retinal spikes during that window may have a somewhat stronger negative influence on relay probability (i.e., push the model to predict “not relayed”) during epochs of heightened activity (Q3 and Q4); however, the magnitude and variability of this effect (as seen in the overlapping CI shading) suggest little qualitative difference between activity levels. On the other hand, filters learned from drifting grating data show a much clearer difference between activity levels, specifically within a time window ∼5 to 20 ms before the target retinal spike (Fig. 8C, inset), such that the filters learned from high activity data (Q3 and Q4) show a faster decay toward zero from the initial positive peak immediately preceding the target retinal spike. This difference suggests a narrowing of the effective integration window of LGN relay cells during epochs of elevated activity. Importantly, this difference is unlikely to be due to differences in the ability of the model to fit the different datasets (Fig. 8*D*), as median paired difference in I_Bernoulli_ between models fit to data from the highest (Q4) and lowest (Q1) activity levels was −0.005 bits/spike (Q4–Q1, MAD 0.041, 95% CI [−0.047, 0.003], *p *≈* *0.353)^g^. Model performance was also not significantly different between Q4 and Q1 subsets for the binary white noise dataset: median paired difference in I_Bernoulli_ was 0.001 bits/spike (MAD 0.008, 95% CI [−0.001, 0.004], *p *≈* *0.396)^h^.

One potential concern with the above analysis is that the data used to train the model differed considerably between quartiles. Although the quartiles are defined based on LGN firing rates, retinal firing rates will of course be highly correlated. Thus, the observed difference in LGN integration dynamics could be due entirely to differences in the training data. To control for this possibility we use a single, fixed filter learned from all the data from a given pair (i.e., the filters shown in Fig. 2) to simulate the relay status of each retinal spike (i.e., the pattern of retinal spikes preceding each target spike is convolved with the learned filter, the output of which is passed through the logistic function and relay status is determined by a coin flip, see Simulating GLMs). We then performed the quartile subsetting and model fitting exactly as for Figure 8. The learned filters for each stimulus type and activity quartile are shown in Extended Data Figure 8-1. Importantly, in this case the training data have exactly the same quartile related differences as for the original analysis, the only difference is that the integration dynamics of the LGN cell are fixed via the simulation. Thus, the fact that the filters learned from all the quartile subsets are highly overlapping suggests that the differences observed in Figure 8*C* are not because of differences in the training data alone. The overlap in the learned filters is especially apparent through the first ∼30–50 ms where the most striking difference in Figure 8*C* can be seen.

10.1523/ENEURO.0088-22.2022.f8-1 Extended Data Figure 8-1 Comparison of RH models fit separately to subsets (quartiles) of simulated data grouped by LGN activity level. The relay status of each retinal spike was determined by simulating a RH GLM with a fixed retinal filter (i.e., the filter did not change with activity level). ***A***, Average retinal filters from RH models fit to each quartile of the binary white noise dataset from low (Q1, green) to high (Q4, purple) based on the activity level of the LGN neuron within a 100-ms period directly preceding the target retinal spike att = 0. Shading represents 95% CI across *N* = 38 pairs. ***B***, Upper, Comparison of model performance (I_Bernoulli_) across all activity subsets. Each dot represents the model performance for a single pair (the spread along the *x*-axis is to aid visualization). ***B***, Lower, Bootstrap estimation of median model performance for each subset. Black dots indicate the median across pairs and black vertical lines indicate the 95% CI of the bootstrap distribution (shown in color, 5000 samples). ***C***, ***D***, Same as ***A***, ***B*** but for the drifting gratings dataset (*N* = 33). Download Figure 8-1, TIF file.

The finding that LGN integration dynamics depend on firing rate proved to be robust to the precise time window used to classify activity levels (tested over a range spanning 50–200 ms; see Extended Data Fig. 8-2*C*,*D*); however, using time windows close to the cycle duration of the drifting grating (i.e., around 250 ms) is likely to produce a severe underestimate of the real difference as it would effectively average over the preferred and nonpreferred phases of the drifting grating (which is the likely cause of the higher variability in firing rate observed during drifting grating stimulation). Consistent with this idea, repeating the analysis using a 250-ms time window to partition the data into quartiles substantially reduced the difference between filters learned from the drifting grating data (filters learned from binary white noise data continued to show no difference; see Extended Data Fig. 8-2*A*,*B*).

10.1523/ENEURO.0088-22.2022.f8-2 Extended Data Figure 8-2 Activity level analysis utilizing different time windows for partitioning retinal spikes. ***A***, RH model filters learned from lowest (Q1) to highest (Q4) activity level subsets for binary white noise data where retinal spike assignment is based on a quartile partitioning of LGN spike count within a 250-ms window preceding each retinal spike. ***B***, Same as ***A*** but for drifting grating data. ***C***, ***D***, Same as ***A***, ***B*** but using a 125-ms window for partitioning. Download Figure 8-2, TIF file.

The awake dataset did not contain a sufficient number of spikes to perform the quartile subsetting procedure that we used for the anesthetized dataset (median number of retinal spikes per-pair in the awake dataset was 2017.0 (MAD 427.5), while anesthetized datasets had a median of 12,303.5 (MAD 5624.0) and 38,425.0 (MAD 18, 150.0) for the binary white noise and drifting grating datasets, respectively). Thus, we used a median split to assign each retinal spike from each pair to a low or high activity subset. The filters learned from low and high subsets showed little difference (Extended Data Fig. 7-2*B*), similar to what was seen in the binary white noise (anesthetized) data although lacking the prolonged negative component (between approximately −90 to −120 ms). Interestingly, the one pair that does appear to show a more substantial difference between filters learned from low and high activity data (pair 200001250) was stimulated with gratings during recording (see Discussion).

**Figure 9.**
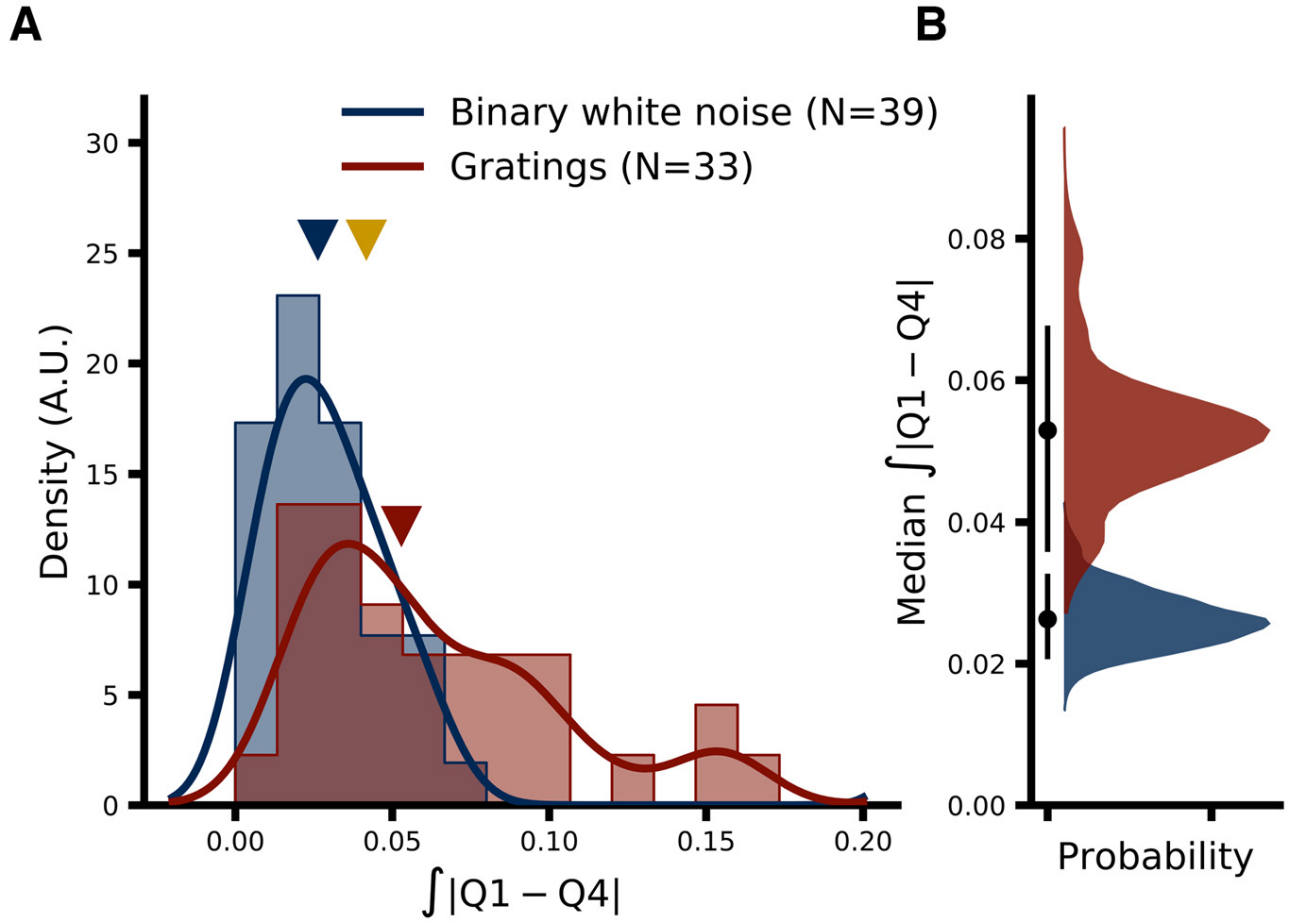
Quantification of differences between filters learned from highest (Q4) and lowest (Q1) activity datasets. ***A***, Population distributions (filled bars) and kernel density estimates (thick lines) of absolute differences between Q4 and Q1 filters for binary white noise (blue) and drifting grating (red) data. Filled triangles denote the median of each distribution. The gold triangle indicates the median difference for the awake dataset for reference (where “high” and “low” were defined by a median split because of fewer spikes in that dataset). ***B***, Estimation of population medians from A. Filled black dots indicate the median and black vertical lines indicate the 95% CI of the bootstrap distributions of population medians shown in blue (red) for binary white noise (drifting grating) data.

To quantify the apparent differences in filters learned from the highest (Q4) and the lowest (Q1) activity data (Fig. 8*C*), we calculated the integral of the absolute difference between the Q1 and Q4 filters for each pair (see Materials and Methods, Classification of retinal spikes by activity level). The distribution of the paired absolute differences, along with kernel density estimates, for each stimulus condition are shown in Figure 9*A* with the corresponding estimation of the median of each distribution show in Figure 9*B*. For the binary white noise dataset the median absolute difference between Q4 and Q1 was 0.024 (MAD 0.010, 95% CI [0.018, 0.028]), and for drifting grating it was 0.055 (MAD 0.026, 95% CI [0.033, 0.067]). For the filters learned from simulated data (Extended Data Fig. 8-1), the median absolute difference was 0.002 (MAD 0.001, 95% CI [0.002, 0.003]) and 0.005 (MAD 0.003, 95% CI [0.003, 0.007]) for binary white noise and drifting grating data, respectively.

A paired permutation test including only pairs for which both binary white noise and drifting grating data were available (*N* = 27) confirmed the differences between the two stimulus conditions: paired median difference (drifting gratings minus binary white noise) in absolute difference was 0.031 (MAD 0.016 95% CI [0.018, 0.039], *p *≈* *0.0002)^i^. Repeating the analysis when only including the 30-ms period preceding the target retinal spike yielded similar results (paired median difference of 0.008, MAD 0.007 95% CI [0.002, 0.012], *p *≈* *0.0004)^j^.

## Discussion

The aim of this study was to investigate how LGN relay cells integrate their retinal inputs over time, and how the integration process changes under different stimulus and network conditions, by using computational models to predict which retinal spikes were relayed on to V1 and which were not. We model retinogeniculate transmission as a coin flip (or Bernoulli) process where the primary quantity of interest is the probability, p, that each incoming retinal spike will be relayed. In the simplest possible model p is a constant given by the mean efficacy across all retinal spikes recorded from a given RGC-LGN cell pair. This constant p model (or homogeneous Bernoulli model) forms the basis of comparison for all other models that we considered, as the constant p model captures the fact that as mean efficacy approaches the extremes (0 or 1) predicting relay status becomes trivial (simply guessing the mean will approach perfect performance). Thus, we chose to quantify model performance in terms of the cross-validated single-spike Bernoulli information ( I_Bernoulli_) which quantifies how informative model predictions are about the relay status of retinal spikes (that were not “seen” during model fitting) relative to a homogeneous model. In our construction, I_Bernoulli_ has units of bits/spike and can take on values between ∼0 and 1, where 0 represents performance no better than a constant p model and 1 represents perfect performance (for details, see Materials and Methods, Assessing model performance).

The fact that I_Bernoulli_ quantifies model performance relative to a homogeneous model is critical given the present context of trying to predict the relay status of retinal spikes. This follows from the fact that the difficulty of predicting relay status varies with mean efficacy: relay status is trivially easy to predict for pairs with a mean efficacy close to zero or one, and is maximally difficult for pairs with a mean efficacy of 0.5. Thus, an optimal performance metric needs to take into account both the quality of the predictions as well as the difficulty of the task for a given pair. I_Bernoulli_ does exactly this. However, as a result the maximum I_Bernoulli_ achievable for pairs with very low or very high mean efficacy is substantially less than one. This fact accounts in part for the low I_Bernoulli_ values achieved by the models considered here, especially on the anesthetized datasets where many pairs have low mean efficacies (9% and 25% of pairs from the drifting grating and binary white noise datasets, respectively, have a mean efficacy<0.05). It should be noted that this behavior is not a deficiency in the I_Bernoulli_ metric, rather it reflects an inherent difficulty in predicting relay status.

We first considered a model where p varies in time according to the elapsed interval since the last retinal spike (ISI) based on extensive evidence that retinal spikes following shorter ISIs are more likely to be relayed because of temporal summation (Usrey et al., 1998; Sincich et al., 2007, 2009; Weyand, 2007; Casti et al., 2008; Wang et al., 2010; Rathbun et al., 2016; Alitto et al., 2019a). Following the framework of Wang et al. (2010), we formalize this observation as a simple model, p=f(ISI)), where the relation between ISI and relay probability (i.e., efficacy) is learned from a subset of the data (training set) and the performance of the model is tested on a separate subset (testing set, see Materials and Methods, Assessing model performance).

We further considered a model where p is a function of the pattern of retinal spikes that an LGN cell receives within a given window of time, what we call the RH model. Conceptually, this can be seen as an extension of the ISI-efficacy model that additionally takes into account the notion that the influence of retinal activity on the current state of a relay cell (i.e., its propensity to relay a retinal input should one arrive) is unlikely to be limited to just the most recent retinal spike. Thus, allowing a model to consider the full pattern of recent spikes from the recorded RGC should improve predictions of relay status and provide a less constrained view of the temporal integration dynamics of retinogeniculate interactions. To that end we used Bernoulli-Logistic GLMs to predict the relay status of each retinal spike based on the convolution of a learned temporal filter (retinal filter) with the pattern of recent retinal activity, the output of which is then mapped to a predicted relay probability (or equivalently, predicted efficacy).

In comparing the parameters learned by the ISI-efficacy and RH models, one critical difference between the models is worth nothing. For the ISI-efficacy model, relay probability is modeled as a univariate, nonlinear function of ISI, while the RH model is a linear function of the multivariate pattern of retinal spikes over a given time window (which is then passed through a logistic nonlinearity). Thus, the similarity of the ISI-efficacy functions and RH retinal filters presented in Figure 2 should be interpreted carefully. However, the rapid decay of both functions does tell a consistent story, namely, that the time windows over which retinal spikes positively interact (i.e., promote a relay probability above the mean) is ∼20–30 ms regardless of the stimulus (gratings or binary white noise) or the state of the animal (anesthetized or awake). This likely accounts for the observation that the RH model only outperforms the ISI-model by the smallest of margins in the anesthetized data (Figs. 6, 7), and not at all in the awake data (though the small size of the awake dataset should be noted).

The final model that we considered was a further augmented version of the RH model that included a second, learned temporal filter (LGN filter) that operated on the recent activity history of the LGN cell, what we call the CH model. As stated previously, for RH models the LGN activity is only used to identify the relay status of each RGC spike, and thus the LGN spike train (and, in particular the LGN spikes not triggered by the recorded RGC) may provide additional information that can help predict the relay status of retinal spikes. While the CH model did outperform the other two models for all datasets tested here, further analysis of the correlates of performance and consideration of the shape of the learned filters suggests that the improvement may be based on different features within the anesthetized and awake datasets. In particular we found that, for the anesthetized dataset the improvement in performance between RH and CH models was correlated with the degree of “burstiness” (i.e., the percentage of LGN spikes that were part of bursts) of the LGN cells of the pairs (Extended Data Fig. 7-2). Furthermore, the shape of the LGN filters, large positive values at very short pretarget-spike latencies, suggests that the model is capturing the increase in retinal efficacy that occurs during geniculate bursts (Alitto et al., 2019b), and this component of the LGN filters was specifically attenuated when noncardinal burst spikes were removed from the data before CH model fitting (Extended Data Fig. 7-1). In contrast, the LGN filters learned from the awake data cannot be accounted for by bursts, as burst were extremely rare in the awake dataset. Instead, the negative component seen between ∼−40 and 0 ms (Fig. 3) likely reflects the influence of a gain control or normalization mechanism that could result from intrathalamic negative feedback through the thalamic reticular nucleus (TRN; or perhaps the longer LGN → V1 → TRN → LGN loop). Across the analyses that we performed, this was the only clear difference between the awake and anesthetized datasets.

Lastly, we asked whether relay cell temporal integration dynamics might differ depending on the level of activity within the retinogeniculate circuit, and whether that difference is seen for both stimulus conditions in the anesthetized data. To that end we assigned each retinal spike to one of four data subsets based on the quartile of LGN activity during the preceding 100 ms (see Materials and Methods, Classification of retinal spikes by activity level) and fit RH models separately to each data subset. We specifically chose to use LGN activity to partition retinal spikes as, although retinal and geniculate activity levels are highly correlated, LGN activity is likely to be more indicative of the activity level of the wider retino-thalamo-cortical circuit. For binary white noise data, learned temporal filters showed little difference between subsets (Fig. 7*A*), while for drifting grating data a substantial difference is observed between ∼5 and 20 ms (Fig. 7*C*) such that filters learned from the highest activity subsets (Q3 and Q4) show a shorter effective temporal integration window (i.e., the duration of time preceding a target spike where the arrival of another retinal spike will increase the likelihood that the target spike is relayed). For the awake dataset, most pairs showed little difference between epochs of higher and lower activity when analyzed in a similar manner (albeit using a simpler median split as there were not enough spikes to reliably fit model to quartile subsets). Interestingly, the one apparent exception (pair 200001250; Extended Data Fig. 7-2*B*) was also the only pair that was stimulated with gratings during recordings. While this is a single example and so should be considered only the slimmest of evidence, it is nonetheless consistent with the idea that the effective integration window of LGN cells, in both the awake and anesthetized states, is dynamically regulated in a manner that is inversely proportional to the ongoing firing rate (i.e., shorter integration windows during periods of higher activity).

While there are several cellular and circuit mechanisms that could underlie the shortening of the temporal integration window, such as spike rate adaptation within relay cells, short-term depression at the retinogeniculate synapse, feedforward inhibition from geniculate interneurons, feedback inhibition (direct or indirectly via cortex) from the thalamic reticular nucleus, or a change in oscillatory activity coming from the retina (Koepsell et al., 2009), the functional consequence of this process is a form a gain control wherein the specificity of geniculate filtering scales with activity level. The idea being that, under lower levels of activity the LGN behaves more permissively and relays patterns of retinal spikes that under higher activity conditions, where the LGN is less permissive, would not be relayed. This process might offer an explanation for several observations about retinogeniculate transmission, such as the finding by Alitto et al. (2019a) that retinal efficacy following ISIs in the ∼5- to ∼25-ms range is higher under low contrast (and thus low activity) than high contrast (and thus high activity) stimulus conditions. Likewise it could potentially explain the finding by Rathbun et al. (2016) that as the contrast of a drifting grating stimulus increases, responses of LGN cells shift to progressively earlier phases of the stimulus cycle and that the rate of this “phase advance” is higher in relay cells compared with their direct retinal inputs. Further work is needed to address whether the magnitude of the integration widow shortening that we observe here quantitatively matches the observations listed above.

### Relationship to previous work

A considerable amount of effort has been put into modeling the computations performed by relay cells of the LGN, due in large part to the fact that simultaneous recordings of both a dominant input (from RGCs) and the output (LGN spiking) is possible. Prior work on modeling retinogeniculate interactions can be coarsely grouped into two approaches: those that focus on LGN processing of retinal spike trains in the absence (Casti et al., 2008; Heiberg et al., 2013), or presence (Norheim et al., 2012) of extraretinal input, and those that include an additional channel for processing the visual stimulus directly (Babadi et al., 2010; Butts et al., 2016). The logic of including the additional stimulus channel is that it enables models to capture stimulus driven effects that are not mediated by the direct retinal input, so that “indirect” effects (e.g., from cortical or TRN feedback) might be uncovered. While this is a powerful approach to studying geniculate computations generally, we instead chose to focus our efforts more narrowly on modeling how LGN cells process individual retinal inputs by trying to predict which retinal spikes were relayed and which were not. This approach is particularly well suited to our data, which consists primarily of recordings of RGC-LGN cell pairs in which the RGC spikes were recorded within the eye. This entails that (1) we can be confident that few, if any, RGC spikes went undetected, and (2) that most of our recordings were made from nondominant RGC inputs. The second point follows from the observation that most relay cells in the cat receive input from two to five RGCs (Cleland et al., 1971; Hamos et al., 1987; Usrey et al., 1999; Martinez et al., 2014), and thus landing an extracellular electrode in the vicinity of the dominant input should be somewhat rare. Conversely, S-potential recordings are likely to reflect just the dominant input (Kaplan and Shapley, 1984; Weyand, 2007). Consistent with this idea, we observed considerably higher mean efficacies in the awake dataset (on average ∼0.52) compared with either the drifting grating (∼0.16) or binary white noise (∼0.1) datasets from the anesthetized animal. Given the above, we reasoned that the most fruitful approach would be to focus on predicting the relay status of the retinal spikes that we did record and avoid making predictions about LGN spikes that were not triggered by the RGC under study.

Overall, this approach emphasizes the computations being performed by relay cells on individual retinal inputs. Previous work has proposed that the core of these computations is well approximated by linear filtering with an exponential kernel (Casti et al., 2008; Heiberg et al., 2013) as suggested by the strong relationship between retinal efficiency and retinal ISI (Usrey et al., 1998; Carandini et al., 2007; Sincich et al., 2007, 2009; Casti et al., 2008; Uglesich et al., 2009; Rathbun et al., 2010; Wang et al., 2010). The strength of taking a statistical approach, as we do here, is that the form of the linear filter is directly learned by the model. Our results confirm that an exponential filter is indeed a good model of relay cell temporal integration and, given the relatively short apparent time constants (on the order of 10–20 ms, consistent with Casti et al., 2008), suggest that the retinal ISI is likely to be the strongest single influence on whether a given retinal spike is relayed or not.

In conclusion, overall, our results suggest that the dominant factor that determines whether or not a given RGC spike is relayed to cortex by the LGN is the retinal ISI, confirming previous findings (Usrey et al., 1998; Carandini et al., 2007; Sincich et al., 2007, 2009; Casti et al., 2008; Uglesich et al., 2009; Rathbun et al., 2010; Wang et al., 2010). However, quantitatively smaller, yet still likely important, contributions were observed for retinal activity further into the past, as well as LGN activity patterns indicative of periods of burst firing. Furthermore, we have demonstrated that the time scale over which the LGN integrates its retinal inputs changes as a function of the level of activity within the retino-thalamo-cortical circuit. This finding raises the possibility that gain control (Shapley and Enroth-Cugell, 1984), a core visual function of the LGN (Alitto et al., 2019a), could be achieved in part by modulating the temporal integration window of LGN relay cells. The source of this modulation remains an open question for future work to explore.

## Synthesis

Reviewing Editor: Niko Busch, Westfalische Wilhelms-Universitat Munster Decisions are customarily a result of the Reviewing Editor and the peer reviewers coming together and discussing their recommendations until a consensus is reached. When revisions are invited, a fact-based synthesis statement explaining their decision and outlining what is needed to prepare a revision will be listed below. The following reviewer(s) agreed to reveal their identity: Robbe Goris.

First off, I am very sorry for the long delay! Both reviews are quite positive about the manuscript. Most of the reviewers' points ask for additional clarifications or elaboration, or for additional control analyses, most (or all) of which should be feasible to do using the existing dataset. I encourage you to carefully address these points.

# Reviewer 1

In this manuscript, Alexander et al investigate temporal filtering within the LGN by modeling the input-output relation of LGN cells. They use data from previously published work (extracellular recordings of monosynaptically connected RGC-LGN cell pairs in anesthetized cats, complemented with a small set of awake recordings in which S-potentials were recorded simultaneously with the LGN spikes they frequently elicit). They ask which spikes from the recorded RGC elicit a spike in their geniculate partner (i.e., are relayed), and which do not. The analysis is built on a clever and rigorous use of the GLM framework. Comparison of different model variants reveals that relay status is primarily determined by the preceding retinal inter-spike interval. Model predictions are slightly improved when the prior activity of the LGN cell is also included (this captures the effects of bursting), and the filter shape exhibits weak but systematic dependency on the level of activity. These results are in line with earlier studies (some of which used the same data) and will not change current thinking about LGN filtering very much. However, the analysis framework is elegant and the dynamic nature of the recovered filters is interesting. I think that this paper is a nice addition to the literature and have no major suggestions for improvement, although I would like to see a model recovery analysis.

Minor suggestion

- The modeling framework is carefully developed as is. However, our confidence in the inferred variables could be increased by adding a recovery analysis in which you simulate the RGC-LGN transformation and show how well the GLM recovers the ground truth. How many spikes do you need to observe to get reasonable error levels? If there are 2-5 RGC inputs and you observe only one of them, how do the non-observed cells impact the inferred filter? Simulations of this nature would further support and strengthen the interpretation of the current analysis.

Typos

Line 155: efficiency, should be efficacy

Line 720: retain, should be retina

# Reviewer 2

This study models the relay of RGC cell spikes by LGN neurons. Defining an RGC spike as relayed if it fell within the time of a short latency peak of a RGC-LGN cross-correlogram, they found that the main factor in their models that was linked with a spike being relayed was the relative timing of the previous RGC spike, consistent with previous work. Their novel findings were that other factors that influenced relay efficacy were earlier LGN spiking activity (particularly bursting) and earlier RGC spiking activity, and that the integration time of RGC to LGN filters got narrower when LGN neuron activity was higher.

Overall, I think this is a good paper with a rigorous approach and some novel findings. However, I think it could do with some improvements in some places which I detail below.

Major points

1. Low J_Bernoulli values for the anaesthetised results

The J_Bernoulli values that assess model performance appear very low for the anaesthetised data, for example being a median of 0.03 for the white noise stimulus and 0.07 for the drifting grating stimulus. Are these low values, they seem low? This limits the interest in the models as the models appear on average not very explanatory. The authors explain that these small values are likely a result of many of the RGCs recorded not being the main RGC that drives the neuron. Also admittedly, some of the individual neurons do have high J_Bernoulli, and the awake results do have relatively high J_Bernoulli. I think some more analysis and discussion is needed to explore this issue. Here are some suggestions

A) Check that the novel results (LGN and RGC spiking influence, high-rate integration narrowing) hold when only the neurons with high J_Bernoulli are used in the analyses done in the paper and in the analyses that I will suggest below. I will leave the authors to define high, I suggest perhaps those neurons J_Bernoulli >0.1 or 0.05, or perhaps the neurons in the top 50%.

B) More details on the distribution of J_Bernoulli. Plot histograms of the distribution of J_Bernoulli for white noise, gratings and awake. Give the means as well as the medians. Mention the highest and lowest J_Bernoulli values seen across neurons.

C) Discuss more whether the J_Bernoulli results are low or not, and if they are low why they are so low and what this means.

D) Get an estimate of the upper bound of J_Bernoulli given neural noise. I am not sure if this is possible, as the paper looks at the mapping from the RGC spikes to the LGN spikes and there are no repeats in the pattern of the RGC spikes. For data where there are repeats there are ways to normalise measures of model performance by an upper bound (Sahani & Linden 2003, Hsu et al. 2004, Schoppe et al. 2016) by estimating the variability in the response to identical inputs. Perhaps there is a way to find the upper bound to these models and data, maybe the authors have some ideas. However, it is fine if the authors cannot do this, just some discussion on how high you might expect the J_Bernoulli to get might be useful (no neurons seem to have J_Bernoulli higher than 0.5).

E) Are S-potentials available from the anesthetised data? It would be good to include a model that uses the S-potentials of the LGN neurons in the anesthetised animals as alternative or additional input to the paired RGC recordings. By this method can the anesthetised results rival the awake results in terms of J_Bernoulli? This would be a valuable addition to the paper, but I recognise it might be a fair amount of work.

2. Only essential model differences

It isn't clear whether the modelling advantage of the CH model over the RH model is due the addition of the LGN history, as there are some other differences between the CH and RH models, such as the use of raised cosine windows. I suggest that the authors run a version of the CH model that is as identical as possible to the full CH model (e.g. with raised cosine window) but without the LGN history, and compare this to the RH model.

Likewise, to check more thoroughly for effects of further back retinal spikes, I suggest the authors run versions of the RH and CH models but with no LGN history and with all RGC spikes set to zero except those used in the ISI model, and then compare to the ISI model.

3. Double checking the filter narrowing isn't an artefact

The statistics of the RGC activity will likely differ between the Q1, Q2, Q3, and Q4 of the LGN activity, and the narrowing to the filter at higher LGN spike rates might be an artefact of the different statistics of the input rather a genuine change in the neural system. While the fact that the J_Bernoulli does not significantly differ between Q1 and Q4 is heartening, I think another check that the results are not an artefact would be valuable. For this I suggest take the original RH model fit shown in Figure 2, and then use this to simulate for all X a binary vector of LGN relay statuses. Then analyse this simulated data, dividing it into the same Q1 to Q4 as the real data. Because in the simulated data the retinal filter does not change, then the Q1 to Q4 filters should not change either. If the Q1 to Q4 filter do narrow for the higher rates for the simulated data, then it is a sign that the result may be at least partially an artefact. The simulation may need to run multiple times. Suggest also running this analysis using the CH model of Figure 3 to provide the simulated data.

I would also be interested to see the filters and equivalent analyses for Q2, Q3 and Q4 for the CH model.

4. Gain control

The authors talk about how the filter narrowing could relate to gain control. It is not clear to me how this is the case. Gain control as I understand it involves adapting to the variance of the input by dividing the neural activity at some point in the system by a quantity such that response becomes the same or similar regardless of the variance. It is not clear to me how the RH or CH model would do division. Perhaps the authors mean adaptation to the mean rather than adaptation to the variance (gain control)? I suggest that the authors either explain more explicitly how the filter narrowing could relate to gain control, or drop mention of gain control.

5. Correlation does not imply causation

This study does not involve experimental manipulation of the system. In a few places the authors imply causation more strongly than they have evidence for, I suggest that they phrase things more carefully in this regard.

6. Illustrations of the models

It would be great to have a figure illustrating the ISI, RH and CH models.

7. Additional main figures

I think Supporting Figure 4 should be made a main figure, as the awake results are the only results with high J_Bernoulli. I also think Supporting Figure 1 should also be a main figure, as this is a sufficiently important result to be mentioned in the abstract.

Detailed/minor points

Line 12. Sinich et al. needs to be in brackets.

100. I assume these are extracellular recordings? please clarify

145. Suggest ‘resulting in’ rather than a semicolon

159. Is L the likelihood or the log likelihood? Sentence needs to be more clear

157. Should subscripts in the equations all be italic. I think typically only variables are italic.

163-166. Explain better

Equations 4 and 5. Should L(y_test) be L(lambdabar_test;y_test)?

188-189. This sentence isn't clear

Equation 6. I am a bit confused here, this doesn't seem consistent with Equation 1 as it depends on P not on t. Please clarify.

210. Should it be bold y that is defined or should it be italic y in Equation 7? Need better definition of what y is.

245. What if you include this time window? No need to do, but please discuss. Could the time of the previous LGN spike relative to the time of current LGN spike be important?

248. Define theta_C

248. Why not raised cosines for the retinal model?

284. ‘where’ not ‘were’ I think.

303. Was every combination of the hyperparameters explored? that is a large space!

345. ‘swapped or not’ with what probability? This is confusing, please explain more clearly.

347. Corrected for what?

411-412. Small bit values. Discuss somewhere that these are small.

Figure 2. Interesting that there doesn't seem to be any or much inhibition, discuss.

499-451. Again small values, please discuss.

500. What % are burst in the anesthetised data? Need to say

507. Is it gain control? The effect of the filter is subtractive rather than divisive in the model. Please elaborate why this can be gain control.

522. Is the normalization reasonable? Exactly what normalization?

534-535. Is this sentence correct? J_Bernoulli for the combined model is 0.26 but it is 0.18 for the ISI model

578. Also give median firing rate standard deviation for gratings and noise separately for context

773-774. Correlation does not equal causation

Figure 7. Suggest change to ‘grouped by LGN activity level’
