# Supplementary figures and images for: Dynamics of Temporal Integration in the Lateral Geniculate Nucleus
Source: eNeuro. 2022 Aug 23;9(4):ENEURO.0088-22.2022. doi: 10.1523/ENEURO.0088-22.2022 (PMC9402337; doi:10.1523/ENEURO.0088-22.2022)

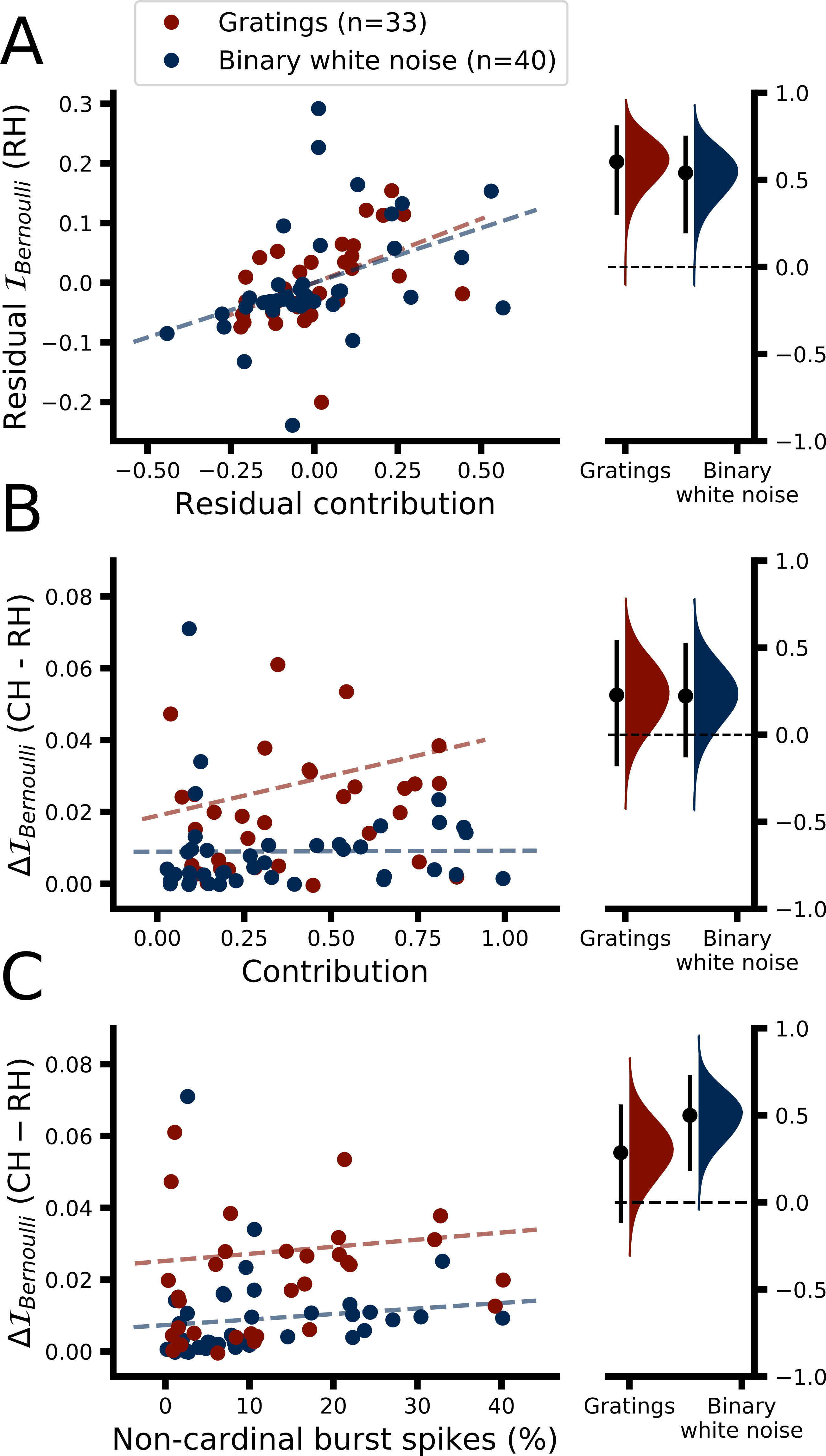

Supplement: Extended Data Figure 7-1 — Correlates of model performance. A, Left, Residual Spearman’s correlation betweenIBernoulli from RH models and retinal contribution where the effect of retinal efficacy on each variable has been removed prior to the analysis. Right, Estimation of correlation coefficient using 5000 bootstrap resamples. Black dots denote point estimates, vertical black lines denote 95% CI, and filled distributions summarize the results of the resampling. B, Left, Spearman’s correlation between model performance improvement (ΔIBernoulli) between CH and RH models and retinal contribution. As retinal efficacy is not correlated with ΔIBernoulli regular Spearman’s correlation was used. Right, estimation analysis for correlation shown at left. C, Left, Spearman’s correlation between ΔIBernoulli and the percent of LGN spikes that were part of identified bursts (using the traditional criteria by Lu et al., 1992) excluding the cardinal spike of each burst. Right, Estimation analysis for correlation shown at left. Download Figure 7-1, TIF file. [file enu-eN-NWR-0088-22-s03.tif]

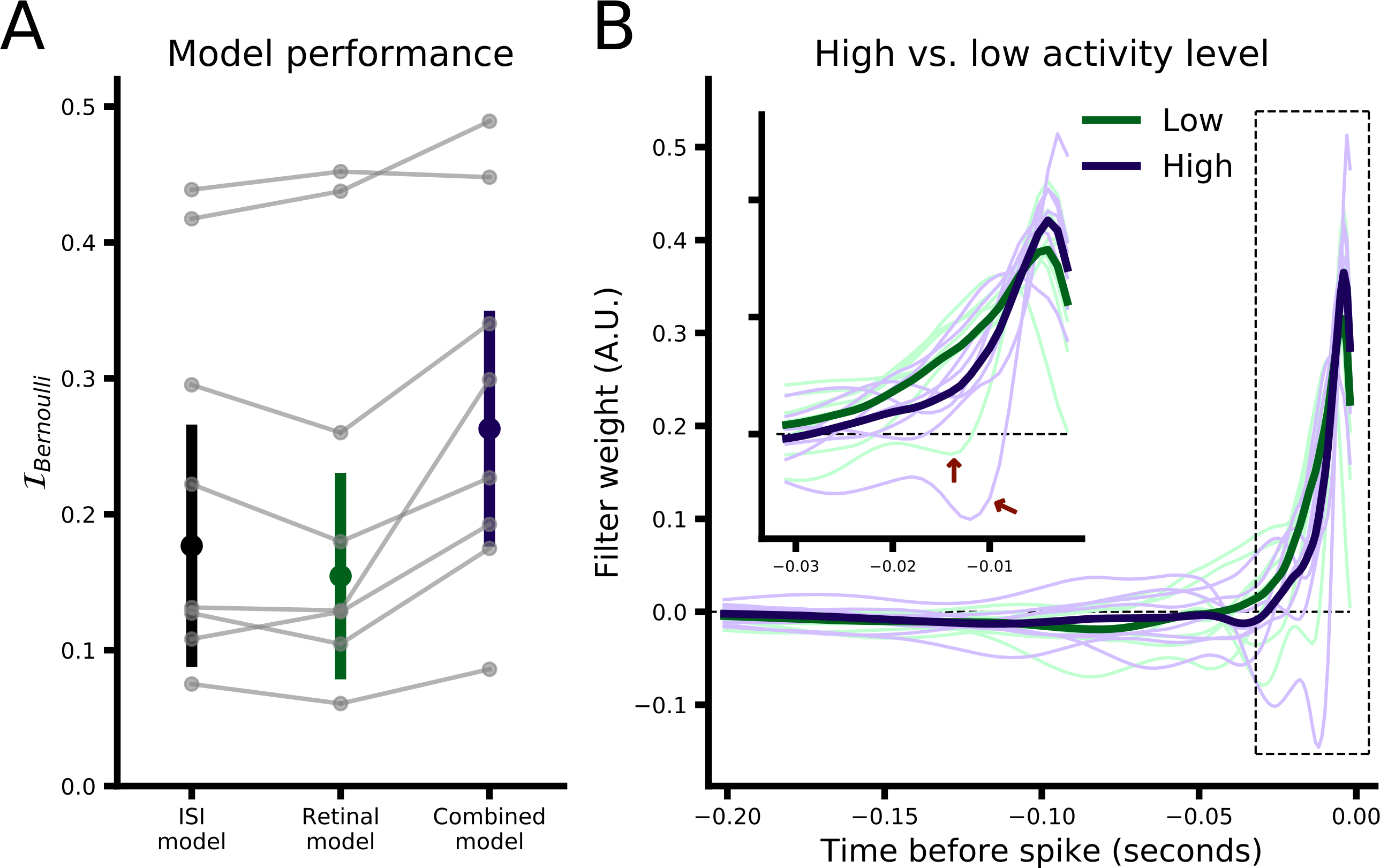

Supplement: Extended Data Figure 7-2 — Model comparison and activity level analysis for awake data. A, Model performance (mean IBernoulli across folds) for each model and pair (grey points) where grey lines connect points that correspond to the same pair. Large, solid color circles indicate the median, and solid-color vertical lines show the MAD, across pairs for a given model (black: ISI model, green: RH model, purple: CH model). B, Retinal filters learned by RH models from low (green) and high (purple) activity datasets (similar to Fig. 6 but using a median split to assign each retinal spike to a dataset). Filters from individual pairs are show in less saturated, thin lines while thick saturated lines indicate the mean across pairs (all filters are scaled to have unit norm to aid visualization). Inset axis highlights the boxed region corresponding to the 30 ms immediately preceding each “target spike” (at t = 0). The red arrows indicate the filters learned from pair 200001250, which is the only pair of the awake dataset that was stimulated with gratings during recording. Download Figure 7-2, TIF file. [file enu-eN-NWR-0088-22-s04.tif]

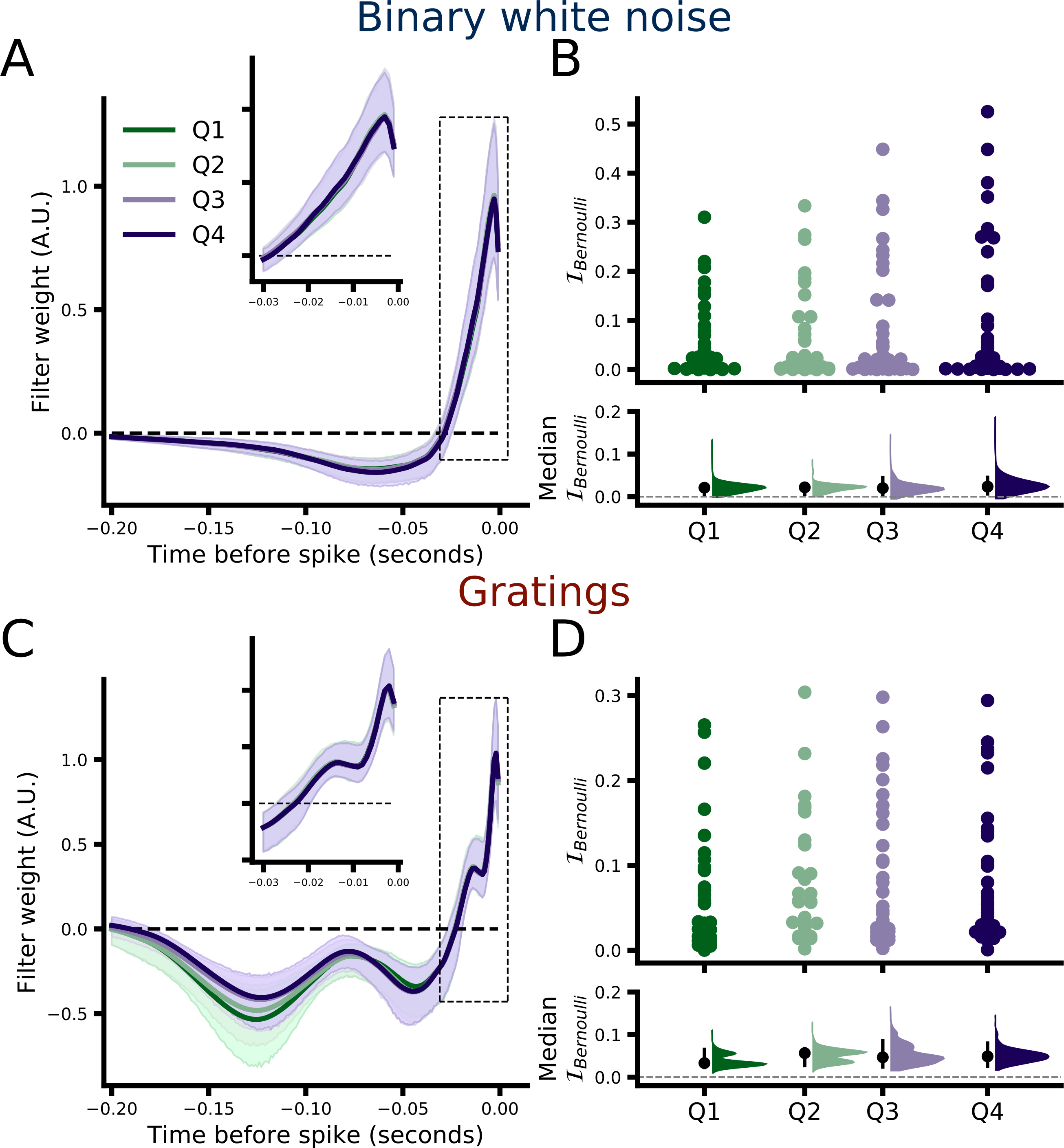

Supplement: Extended Data Figure 8-1 — Comparison of RH models fit separately to subsets (quartiles) of simulated data grouped by LGN activity level. The relay status of each retinal spike was determined by simulating a RH GLM with a fixed retinal filter (i.e., the filter did not change with activity level). A, Average retinal filters from RH models fit to each quartile of the binary white noise dataset from low (Q1, green) to high (Q4, purple) based on the activity level of the LGN neuron within a 100-ms period directly preceding the target retinal spike att = 0. Shading represents 95% CI across N = 38 pairs. B, Upper, Comparison of model performance (IBernoulli) across all activity subsets. Each dot represents the model performance for a single pair (the spread along the x-axis is to aid visualization). B, Lower, Bootstrap estimation of median model performance for each subset. Black dots indicate the median across pairs and black vertical lines indicate the 95% CI of the bootstrap distribution (shown in color, 5000 samples). C, D, Same as A, B but for the drifting gratings dataset (N = 33). Download Figure 8-1, TIF file. [file enu-eN-NWR-0088-22-s05.tif]

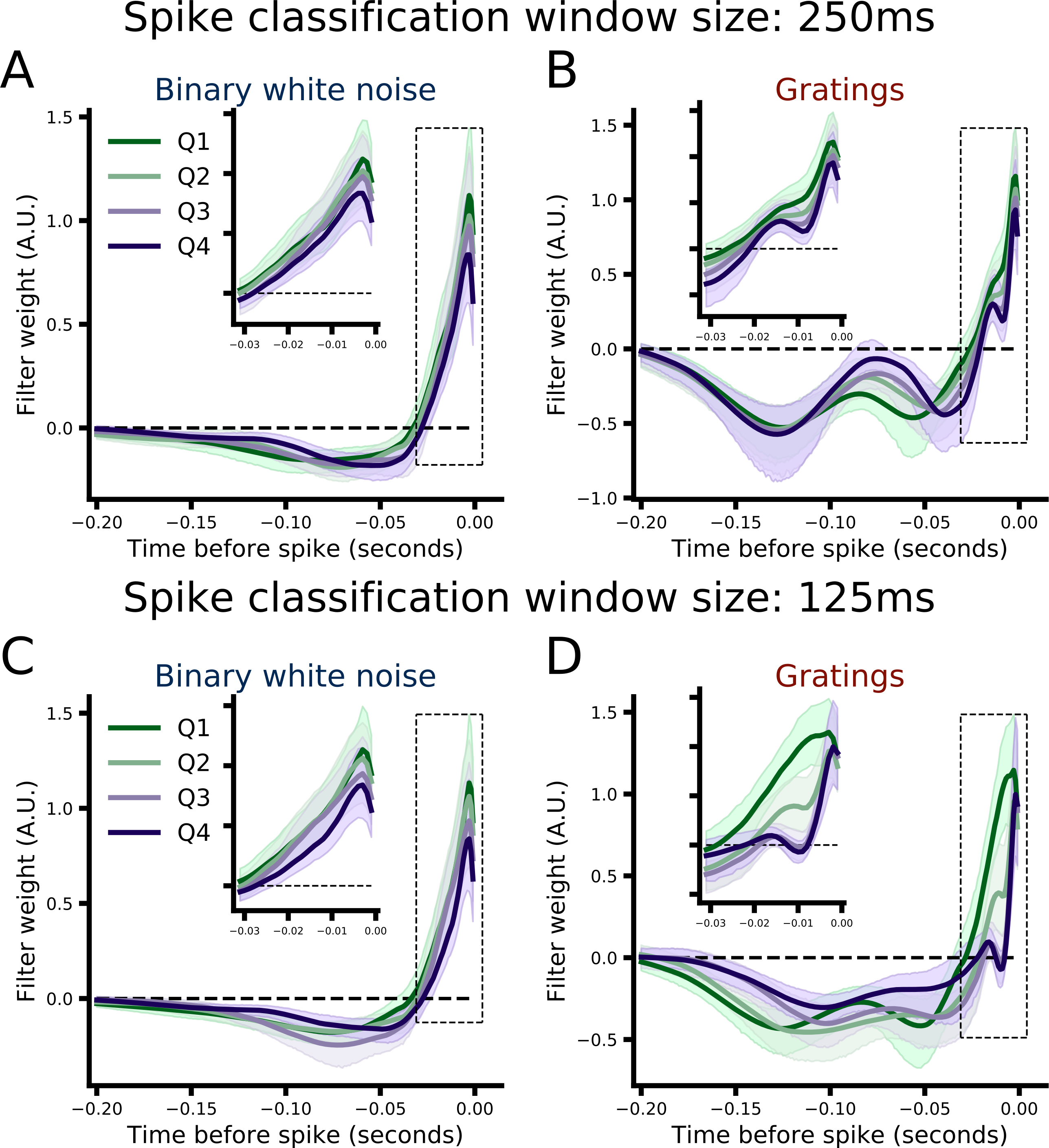

Supplement: Extended Data Figure 8-2 — Activity level analysis utilizing different time windows for partitioning retinal spikes. A, RH model filters learned from lowest (Q1) to highest (Q4) activity level subsets for binary white noise data where retinal spike assignment is based on a quartile partitioning of LGN spike count within a 250-ms window preceding each retinal spike. B, Same as A but for drifting grating data. C, D, Same as A, B but using a 125-ms window for partitioning. Download Figure 8-2, TIF file. [file enu-eN-NWR-0088-22-s06.tif]
